# Highly Thermal Conductive and Electromagnetic Shielding Polymer Nanocomposites from Waste Masks

**DOI:** 10.1007/s40820-025-01796-z

**Published:** 2025-05-20

**Authors:** Xilin Zhang, Wenlong Luo, Yanqiu Chen, Qinghua Guo, Jing Luo, Paulomi Burey, Yangyang Gao, Yonglai Lu, Qiang Gao, Jingchao Li, Jianzhang Li, Pingan Song

**Affiliations:** 1 State Key Laboratory of Efficient Production of Forest Resources and MOE Key Laboratory of Wood Material Science and Application, Beijing Forestry University, Beijing, 100083 People’s Republic of China; 2 College of Materials Science and Engineering, Nanjing Forestry University, Xuanwu District, Longpan Road 159, Nanjing, 210037 People’s Republic of China; 3 Centre for Future Materials, School of Agriculture and Environmental Science, University of Southern Queensland, Springfield, 4300 Australia; 4 State Key Laboratory of Organic-Inorganic Composites, Beijing University of Chemical Technology, Beijing, 100029 People’s Republic of China

**Keywords:** Mask waste, Repurposing, Thermal conductivity, Electromagnetic interference shielding, Life cycle assessment

## Abstract

Over 950 billion (about 3.8 million tons) masks have been consumed in the last four years around the world to protect human beings from COVID-19 and air pollution. However, very few of these used masks are being recycled, with the majority of them being landfilled or incinerated. To address this issue, we propose a repurposing upcycling strategy by converting these polypropylene (PP)-based waste masks to high-performance thermally conductive nanocomposites (PP@G, where G refers to graphene) with exceptional electromagnetic interference shielding property. The PP@G is fabricated by loading tannic acid onto PP fibers via electrostatic self-assembling, followed by mixing with graphene nanoplatelets (GNPs). Because this strategy enables the GNPs to form efficient thermal and electrical conduction pathways along the PP fiber surface, the PP@G shows a high thermal conductivity of 87 W m⁻^1^ K⁻^1^ and exhibits an electromagnetic interference shielding effectiveness of 88 dB (1100 dB cm^−1^), making it potentially applicable for heat dissipation and electromagnetic shielding in advanced electronic devices. Life cycle assessment and techno-economic assessment results show that our repurposing strategy has significant advantages over existing methods in reducing environmental impacts and economic benefits. This strategy offers a facile and promising approach to upcycling/repurposing of fibrous waste plastics.

**Supplementary Information:**

The online version contains supplementary material available at 10.1007/s40820-025-01796-z.

## Highlights

- Fabricating low-cost, high-performance, scalable polypropylene (PP)@graphene (G) nanocomposites from recycled PP fibers in waste masks by a simple electrostatic self-assembly hot-pressing method.
- The resultant PP@G presents a high thermal conductivity of 87 W m^−1^ K^−1^ and a high electromagnetic interference shielding effectiveness of 88 dB (1100 dB cm^−1^).

**Supplementary Information:**

The online version contains supplementary material available at 10.1007/s40820-025-01796-z.

## Graphical abstract

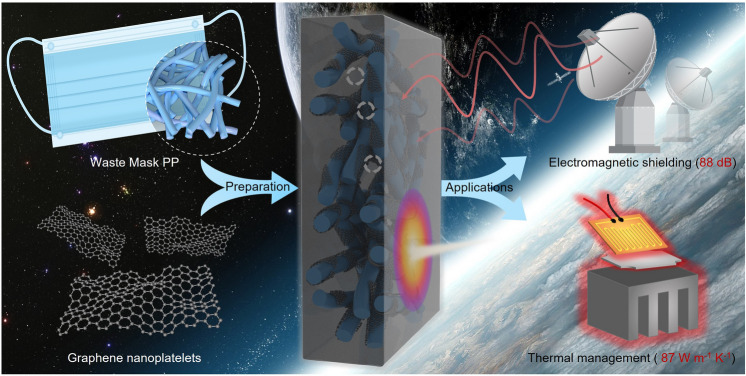

## Introduction

**Fig. 1:**
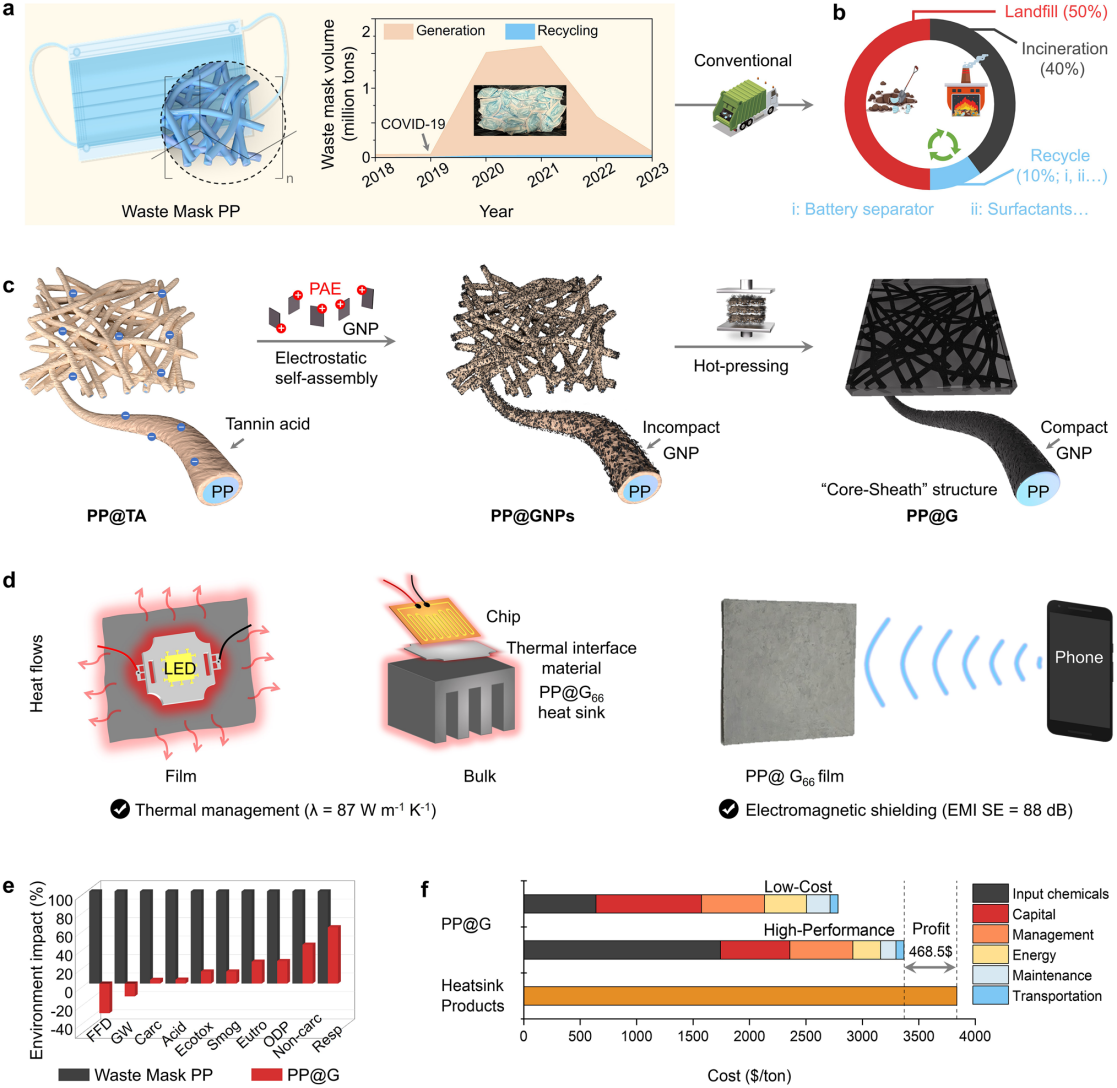
**a** Estimation of waste masks volumes and reuse data from 2018 to 2023 [4]. **b** Conventional route for disposing waste masks. **c** Preparation process of highly thermal conductive nanocomposites with EMI shielding property from waste mask PP. **d** Schematic illustration of as-prepared PP@G nanocomposites for thermal management and photograph of the PP@G nanocomposites for EMI shielding applications. **e** Life cycle assessment of 1 kg PP: comparing landfill disposal versus PP@G nanocomposite preparation: Fossil fuel depletion (FFD); Global warming (GW); Carcinogenics (Carc); Acidification (Acid); Ecotoxicity (Ecotox); Smog; Eutrophication (Eutro); Ozone depletion (ODP); Non carcinogenics (Non-carc); Respiratory effects (Resp). **f** Techno-economic analysis (TEA) of as-prepared low-cost (low GNP contents) and high-performance (high GNP contents) PP@G nanocomposites in the **c** process

The frequent outbreaks of epidemic respiratory diseases have led to a significant increase in the use of disposable medical masks, primarily composed of polypropylene (PP), which accounts for more than 90% by weight [1–3]. Recent statistics indicate that due to the COVID-19 outbreak, the global consumption of waste masks has exceeded 950 billion units (about 3.8 million tons) in the last four years (Fig. 1a) [4]. Most of these waste masks are disposed of through incineration or landfill. Incineration of PP releases toxic gases and organic pollutants, such as dioxins and furans, contributing to air pollution [5–7]. Landfilled PP takes hundreds of years to fully degrade, generating large amounts of microplastics that cause long-term pollution of water sources, soil, and food chains [8–10]. Therefore, it is imperative to find cost-effective methods to convert PP from waste masks into high-performance products and extend the lifecycle of PP materials to reduce their environmental impacts [11].

In recent years, researchers have explored some recycling methods of waste masks, such as using PP from waste masks as raw materials to prepare surfactants, microwave absorbing materials, and battery separators (Fig. 1b). For example, Xu et al. employed a temperature gradient pyrolysis method combined with oxidation and saponification to produce high-value surfactants from PP [12]. Yu et al. deposited Ni(OH)₂ on waste masks and carbonized them at 700 °C to create CNTs/Ni hybrid materials with excellent microwave absorption capabilities [13]. Guo et al. coated PP with hydrogenated nanocellulose (hNCNC) and aluminum nitride (AlN) to prepare multifunctional Janus battery separators, enabling lithium-selenium batteries to achieve outstanding high-rate performance and excellent cycling stability [14]. While these studies provide possibilities for the reutilization of waste masks, they still face numerous challenges in practical applications, such as requiring high-temperature pyrolysis or complex carbonization processes, low mechanical strength, and suboptimal performance of target products. Therefore, it remains a great challenge to develop a cost-effective and large-scale recycling strategy for these waste masks.

Meanwhile, the development of high-power and highly integrated electronic devices in recent years has created an urgent demand for advanced polymer-based thermal management materials (TMMs) [15–18]. In this context, we hypothesize that PP fibers in waste masks can be used as a polymer fibrous matrix to prepare TMMs due to their low cost, ease of processing, and high aspect ratio [19–22]. Therefore, this upcycling process can not only solve the waste masks problem, but also provide new insights for the development of advanced TMMs.

In this study, we aim to repurpose waste masks to fabricate PP-based TMM nanocomposites (PP@G) with high electromagnetic interference (EMI) shielding performance by assembling thermally conductive filler graphene nanoplatelets (GNPs) on tannic acid (TA)-decorated PP fibers (Fig. 1c). The as-prepared PP@G shows a high thermal conductivity (TC) (87 W m⁻^1^ K⁻^1^) and a high electromagnetic interference shielding effectiveness (EMI SE) (88 dB) (1100 dB cm^−1^). The PP@G nanocomposites hold great potential for heat dissipation and EMI shielding applications (Fig. 1d). In addition, life cycle assessment (LCA) and techno-economic assessment (TEA) are also performed to evaluate the advantages of this upcycling strategy over existing methods [23, 24]. This work paves the way for a new upcycling strategy for waste masks and other plastics waste, contributing to creating a sustainable environment and circular economy.

## Experimental Section

### Materials

Graphene nanoplatelets (GNPs, KNG-G2) were provided by Xiamen Knano Graphene Technology Co., Ltd. (China). Polyamide-epichlorohydrin resin (PAE, 12.5 wt%) was purchased from Shandong Tiancheng Chemical Co., Ltd. (China). Tannic acid (TA) was provided by Tianjin Heowns Biochem Technologies Co., Ltd. (China). Waste masks were obtained from municipal solid waste.

### Preparation of the PP@G Nanocomposites

TA of 50 mg mL^−1^ and PAE of 25 mg mL^−1^ under neutral pH conditions were selected as the optimal assembly parameters (Fig. S1). 1 g of GNPs was dispersed in 10 g of PAE aqueous solution (25 mg mL^−1^) via ultrasonication (750 W, 20 kHz) for 30 min to obtain PAE@GNPs dispersion. Meanwhile, waste masks were treated with NaOH solution (1 mol L^−1^) for 1 h at 25 °C, followed by washing with deionized water to neutrality. The PP nonwoven fabric (0.4 g) extracted from washed masks was cut into small pieces (2 mm × 2 mm) and immersed in 50 g of TA solution (50 mg mL^−1^) for 1 h with stirring (300 r min^−1^) at 25 °C. After filtration, the obtained PP@TA was mixed with PAE@GNPs dispersion via ultrasonication (750 W, 20 kHz) for 30 min. The mixture was then filtered and dried in a vacuum oven at 60 °C for 12 h to obtain PP@GNPs. Finally, the PP@G nanocomposites were fabricated by hot-pressing the PP@GNPs at 140 °C and 50 MPa for 30 min. Additionally, the GNPs and the PP nonwoven fabric were mixed at specific mass ratios (0.1:0.4, 0.2:0.4, 0.4:0.4, 0.8:0.4) and used to fabricate PP@G nanocomposites with varying GNP content following the same method as described above.

### Statistical Analysis

All experimental representations were carried out in parallel for three times, and the data were processed in Excel software, here represented by mean ± standard deviation. The difference was statistically significant as determined by one-way analysis of variance (*P* < 0.05).

## Results and Discussion

### Preparation and Characterization of PP@G Nanocomposites

To prepare high-performance PP-based TMMs based on GNPs, it is critical to ensure a good interfacial compatibility. To achieve this, TA with a catechol structure was chosen to treat the surface of PP fibers from waste masks to form PP@TA, because the catechol groups in TA can have strong π–π stacking interactions with GNPs [25]. Meanwhile, GNPs were uniformly dispersed in an aqueous solution of polyamide-epichlorohydrin resin (PAE) via cation–π interactions to prepare a positively charged stable PAE@GNPs solution. Then, the target PP@G nanocomposites were prepared by mixing PP@TA and PAE@GNP via electrostatic self-assembling, filtration and drying, and hot-pressing (Figs. 1c and S2).

Subsequently, large-sized (170 mm × 170 mm) PP@G nanocomposites can be achieved under laboratory conditions (Fig. S2), and could be scaled up to over 1000 mm × 1000 mm according to the size of hot-press machine in industrial settings. The resulting PP@G gives a high TC of 87 W m⁻^1^ K⁻^1^ and a high EMI SE of 88 dB (1100 dB cm^−1^) (Fig. 1d).

Additionally, the PP@G nanocomposite exhibits a high flexural stress of 45 MPa and an acceptable tensile stress of 14 MPa (Fig. S3). After exposure to 100 °C for 24 h and water immersion for 24 h, the PP@G nanocomposites demonstrated significant environmental resistance, maintaining 80% and 50% of their original mechanical strength, respectively, confirming their suitability for applications in harsh environments (Fig. S4). The peeling tests are carried out to investigate the interfacial bonding strength of the PP@G nanocomposites under external force. As shown in Fig. S5, the prepared PP@G nanocomposite exhibits a high peeling force of 970 N and a peeling stress of 2.3 MPa, indicating strong interfacial bonding, which enhances the durability and long-term performance of the PP@G nanocomposites.

The LCA reveals that the PP@G nanocomposite prepared by 1 kg PP significantly reduces environmental impacts compared to the landfill method, especially in terms of fossil fuel depletion (−3.47 vs 10.80 MJ), global warming potential (−0.351 vs 2.53 kg CO_2_ eq), and ecotoxicity (1.02 vs 7.81 CTUe). The negative values observed in fossil fuel depletion and global warming potential categories demonstrate that the PP@G preparation process offers environmental benefits through the upcycling/repurposing of PP materials (Fig. 1e and Table S1). TEA was conducted to compare the PP@G heat dissipation material with commercial products. The assessment indicates that repurposing 1 ton of waste masks to produce PP@G products could yield a profit of approximately $468.50 compared to equivalent commercial heat dissipation materials (Fig. 1f). Thus, this upcycling strategy is economically feasible.

**Fig. 2:**
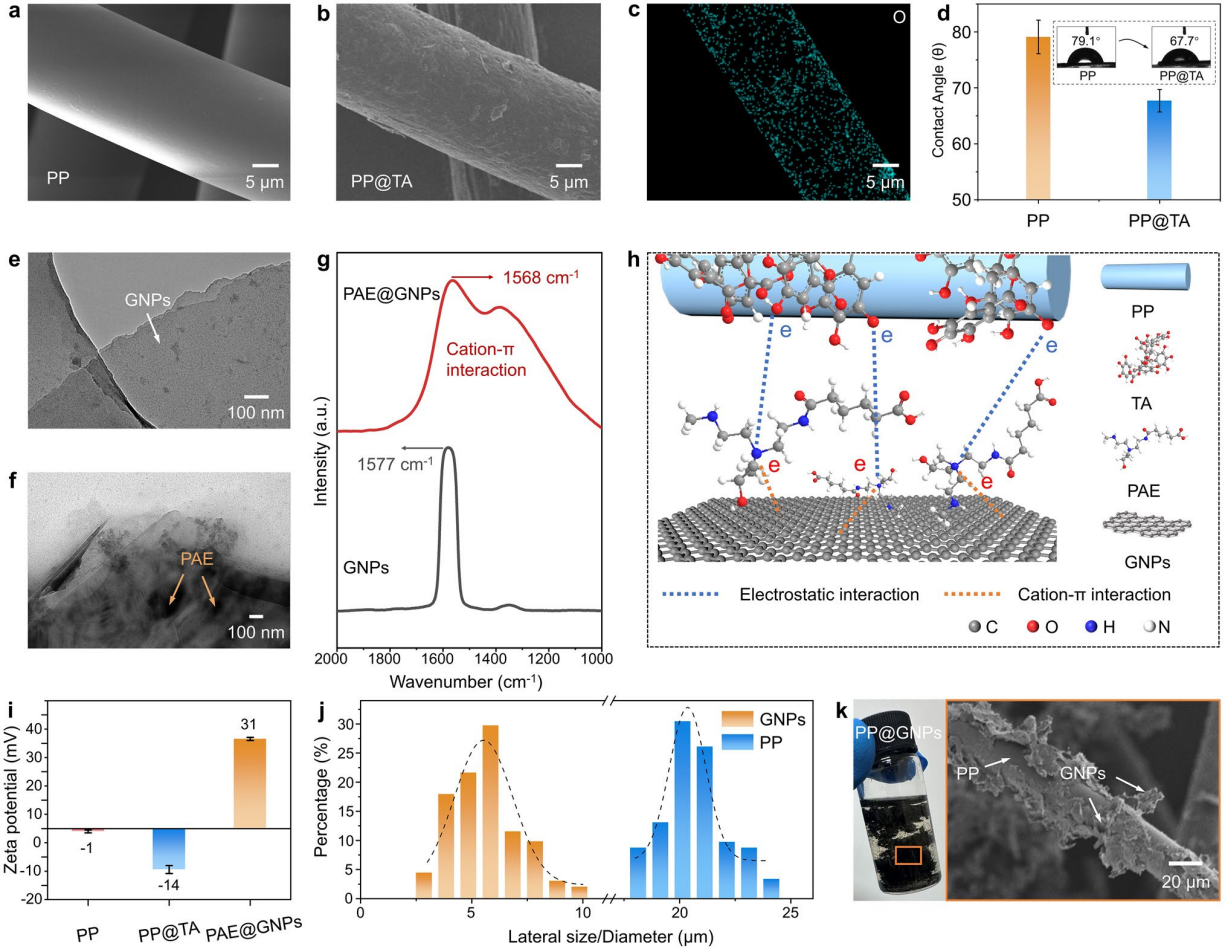
SEM images of **a** PP fiber and **b** PP@TA fiber; **c** EDS elemental maps of O distribution on PP@TA. **d** Contact angles of PP fiber and PP@TA. TEM images of **e** GNPs and **f** PAE@GNPs. **g** Raman spectra of GNPs and PAE@GNPs. **h** Schematic illustration of assembly process of PP@TA and PAE@GNPs. **i** Zeta potentials of PP fiber, PP@TA, PAE@GNPs in water. **j** Lateral size distributions of GNPs and diameter distributions of PP fibers. **k** Photograph and SEM image of PP@GNPs

Compared to smooth surfaces of the PP fibers (Fig. 2a), the scanning electron microscopy (SEM) revealed that the TA-modified PP fiber surface became wrinkled and much rougher (Fig. 2b), indicating successful loading of TA onto the PP fiber surface. Additionally, energy-dispersive spectroscopy (EDS) analysis showed a clear outline of PP fiber in the oxygen element scan (Fig. 2c), indicating uniform distribution of TA on the PP fiber surface. Furthermore, the contact angle of the TA-modified PP fiber decreased from 79.1° to 67.7° (Fig. 2d), demonstrating significantly increased hydrophilicity of PP@TA. These results confirm the uniform attachment of TA on the PP fiber surface, providing an excellent fiber matrix material for the preparation of polymer-based TMMs.

Transmission electron microscopy (TEM) images (Fig. 2e) showed that GNPs exhibited a distinct layered structure with smooth surfaces. The prepared PAE@GNPs maintained the layered structure, but black regions of PAE molecular aggregates appeared on the surface (Fig. 2f). This morphological change indicates successful modification of GNPs by PAE. Raman spectroscopy analysis showed a 9 cm⁻^1^ red shift in the G band of PAE@GNPs compared to GNPs (Fig. 2g), confirming the occurrence of cation-π interactions between PAE and GNPs [26]. Because of these strong cation-π interactions, the PAE@GNPs forms a uniform solution which can be stable for more than 48 h (Fig. S6), indicating that PAE modification significantly enhances the water dispersibility and stability of GNPs.

After TA modification of PP fiber, zeta potential tests showed that the potential of PP@TA decreased from –1 (the potential of PP) to –14, indicating that PP@TA carries a negative charge (Fig. 2i). Consistent with predictions, the potential of PAE@GNPs was + 31, indicating a positive charge (Fig. 2i). When PAE@GNPs and PP@TA are mixed, the positively charged PAE@GNPs are adsorbed onto the negatively charged PP@TA through electrostatic interactions, forming PP@GNPs, as illustrated in the model mechanism diagram (Fig. 2h). To further reveal the electrostatic self-assembly mechanism between PP@TA and PAE@GNPs, we mixed TA and PAE as model substances, which exhibited significant self-assembly behavior, transitioning from a transparent state to a uniformly dispersed emulsion state (Fig. S7a). Furthermore, XPS N 1*s* spectrum analysis shows that the N⁺-C peak of PAE shifts from 401.5 to 401.9 eV (Fig. S7b). The results indicate that the electrostatic self-assembly behavior originates from the electrostatic interaction between hydroxyl groups in TA and azetidinium groups in PAE (Fig. S7c) [27].

Generally, attaching thermally conductive fillers to polymer fiber matrices with larger diameters is conducive to constructing efficient thermal conduction pathways. Therefore, the sizes of PP fibers and GNPs were measured, as shown in Figs. 2j and S8. The results indicated that the average lateral size of GNPs and the diameter of PP fibers were approximately 6 and 20 μm, respectively, which means that the PP fiber provides sufficient space for effective attachment of GNPs. Compared to the PP fiber, the surface of PP@GNPs exhibited a distinct coating of deposited GNPs (Fig. 2k). As the GNP content increased, this coating completely covered the PP fiber surface, forming a “core-sheath” structure (Figs. S9 and S10), where the PP fiber serves as the “core” and GNPs form a continuous “sheath”.

### Thermal Conductivity and Thermal Management Performance

**Fig. 3:**
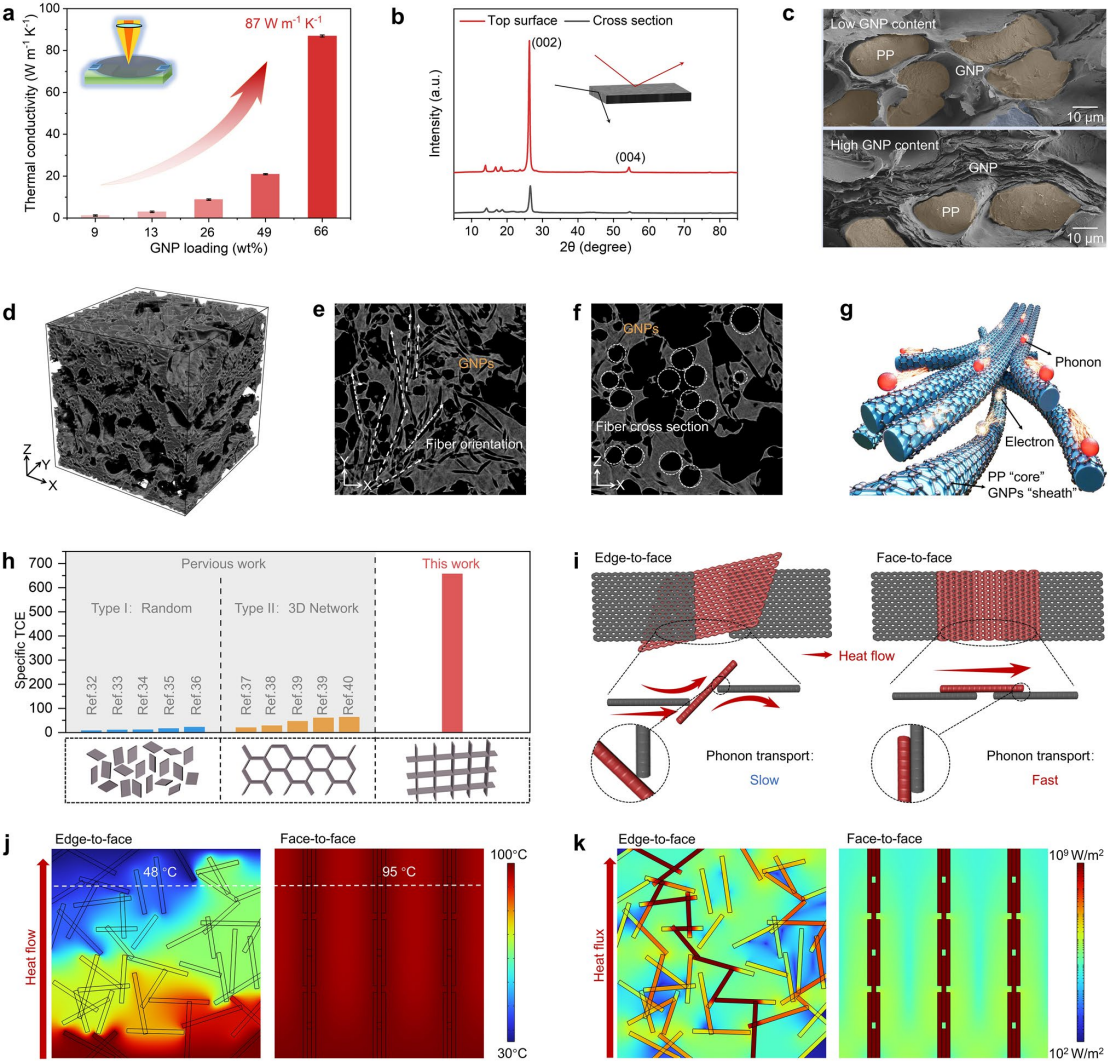
**a** Changes in the in-plane TC of as-prepared PP@G nanocomposites with varying GNP contents. **b** XRD spectra of as-prepared PP@G nanocomposite as measured on the top surface and cross-section (inset shows directions of incident X-rays). **c** SEM images of the cross-sections of as-prepared PP@G nanocomposites with low and high GNP content. **d** 3D nano-CT image of 1 mm long as-prepared PP@G nanocomposite and the corresponding **e** X–Y and **f** X–Z edge plane. **g** Schematic illustration of the interconnected GNPs network that forms efficient phonon/electron transport channels within as-prepared PP@G nanocomposite. **h** Comparison of in-plane specific TCE between as-prepared PP@G_66_ nanocomposites and other previously reported filler-filled polymer-based nanocomposites with random and 3D networked nanofiller structures. Comparison of **i** two GNPs distributions (edge-to-face and face-to-face) and the corresponding **j** heat flow and **k** heat flux

To characterize the TC of PP@G nanocomposites, the effect of different GNP contents on the in-plane TC was first tested. The corresponding TC was calculated using Fourier's law [28] (Eq. 1). As shown in Fig. 3a, when the GNP contents increased from 9 to 66 wt% (Fig. S11), the TC of PP@G nanocomposites increased from 1.2 to 87 W m⁻^1^ K⁻^1^, showing that the TC of PP@G nanocomposites is proportional to the GNP contents, because the increased GNP contents form a more continuous and efficient thermal conduction pathway. Notably, the high TC of PP@G nanocomposites with 66 wt% GNPs (PP@G_66_) surpasses the values of most reported polymer-based TMMs (Fig. S12 and Table S2). Additionally, the steady-state heat flow method (ASTM D5470) was also used to measure the TC, which was close to the out-of-plane TC values determined by the laser flash method (Table S3), which also validates the accuracy of the in-plane TC of the PP@G nanocomposites obtained using the laser flash method.

To better understand the thermal conductivity effect of GNPs in PP@G nanocomposites, two classical theoretical models [29] were used to predict the TC values of PP@G nanocomposites (Table S4) and compared with the experimental values. As shown in Fig. S13, the TC values of PP@G nanocomposites lie between the Agari and Maxwell-HS^+^ models, indicating part of GNPs are parallelly attached on the PP fiber. To further investigate the orientation of GNPs in the PP@G nanocomposites, the structure of PP@G nanocomposites was characterized using 2D wide-angle X-ray scattering (WAXS). As shown in Fig. S14, the innermost halo corresponds to the (002) plane of GNPs, presenting an incomplete closed arc, indicating significant in-plane orientation of GNPs in the nanocomposites [30]. Furthermore, 1D X-ray diffraction (XRD) analysis shows that when the X-ray incident direction is perpendicular to the GNPs orientation, distinct diffraction peaks of graphite's (004) and (002) planes appear at 54.7° and 26.6° [31]; these characteristic peaks significantly weaken or disappear when the X-ray is incident parallel to the GNPs orientation (Fig. 3b). These results provide further evidence for the orientation of GNPs in the nanocomposites.

GNPs form a highly ordered, continuous, and efficient phonon/electron transport channel structure along the PP fiber surfaces under pressure, promoting rapid heat transfer and thereby significantly enhancing the TC of the PP@G composites (Fig. 3c-g). To investigate the contribution of the highly oriented GNPs to the TC of PP@G, the specific thermal conductivity enhancement (TCE) of PP@G nanocomposites was compared with the other thermally conductive polymer-based nanocomposites (Fig. 3h, Eq. S2, and Table S5). The distribution morphology of thermally conductive filler in the nanocomposites can be categorized into two types: Type I (Random), where the thermally conductive filler is randomly dispersed within the matrix, resulting in large contact thermal resistance (R_c_) with the polymer matrix and displaying a lower specific TCE; Type II (3D Network), where the thermally conductive filler forms a 3D interconnected network, showing better specific TCE, but with weak in-plane orientation. Unlike these two microstructures, the prepared PP@G nanocomposites have a highly oriented structure with face-to-face contacted GNPs, which achieves a high specific TCE of 660 in the PP fiber direction, which is a record value compared to polyolefin-based thermally conductive nanocomposites [32–40]. Additionally, the production costs of PP@G nanocomposites are lower than the existing thermally conductive polymer-based nanocomposites (Fig. S12).

The Rc tests were conducted on nanocomposites with different filler distributions. The PP@G shows a R_c_ value of 10^4^ K W⁻^1^, which is two orders of magnitude smaller than that of Type I (≈10^6^ K W⁻^1^) and one order of magnitude smaller than that of Type II (≈10^5^ K W⁻^1^) (Figs. S15 and S16). This is largely attributed to two factors: (I) GNPs “sheath” form continuous and uniform heat conduction pathways along PP fiber “core” surfaces, significantly reducing the thermal resistance at the filler-matrix interface; and (II) the face-to-face oriented GNPs reduce phonon scattering between graphene nanoplatelets. Finite element analysis (FEA) was used to visually verify the thermal conduction advantages of oriented filler distribution compared to random filler distribution, as shown in Fig. 3i–k. The results indicate that, within the same simulation time, oriented filler distribution achieves not only a higher upper temperature but also a more uniform temperature distribution compared to the random filler distribution.

**Fig. 4:**
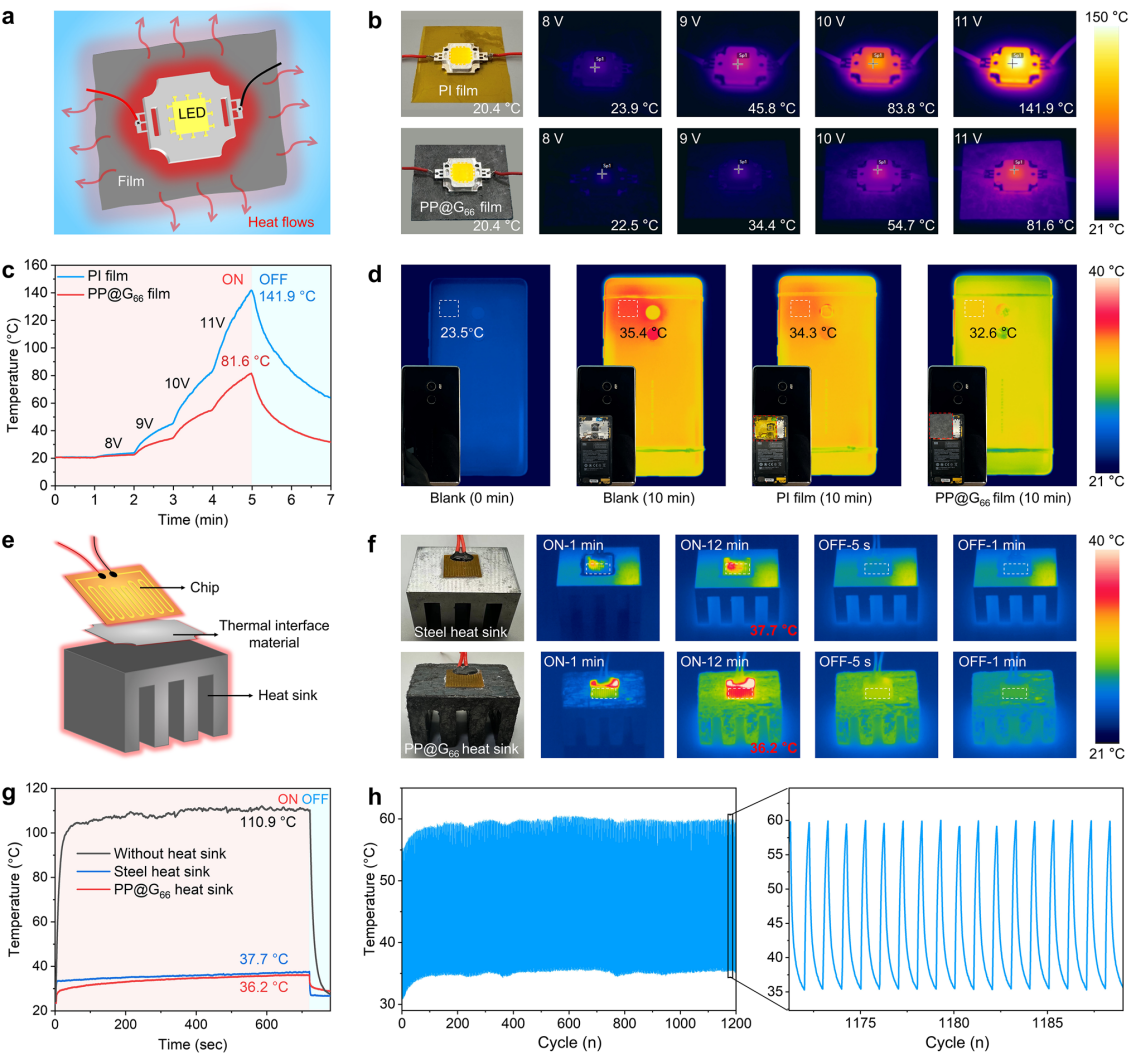
**a** Schematic illustration of heat transfer from LED lights using PI film or as-prepared PP@G_66_ film as cooling substrate. **b** Thermal infrared images of LED lights using as-prepared PP@G_66_ film and PI film as cooling substrate at different voltages, respectively, and **c** corresponding temperature changes. **d** Thermal infrared images of smartphones integrated with as-prepared PP@G_66_ film and PI film. **e** Schematic diagram of heat transfer from flexible circuit using steel heat sink or as-prepared PP@G_66_ heat sink as cooling substrates. **f** Thermal infrared images of flexible circuit with integrated steel heat sink and as-prepared PP@G_66_ heat sink at different voltages and **g** corresponding temperature changes. **h** Cyclability of flexible circuit with as-prepared PP@G_66_ heat sink

To evaluate the thermal management efficiency of PP@G nanocomposites as TMMs, we prepared both film and block types PP@G_66_ nanocomposites to serve as cooling substrates and heat sinks for electronic devices. PP@G_66_ film was used as a cooling substrate for LED lights. Compared to the commercial PI cooling substrate, the PP@G_66_ film demonstrates better heat dissipation capacity (Fig. 4a–c). For example, when a 12 V voltage was applied, the LED light temperature with the PP@G_66_ film was nearly 60 °C lower than that with the commercial PI film. This is because of the much higher TC value of PP@G_66_ than PI (0.2 W m^−1^ K^−1^). Additionally, the PP@G_66_ film exhibits better heat dissipation performance than PI film on smartphone (Fig. 4d). After 10 min of high-load operation, both the smartphone with the commercial PI film and the one without any film exhibited local overheating. The temperatures rose from 23.5 to 34.3 and 35.4 °C, respectively, with heat concentrated in the CPU area and unable to dissipate quickly. In contrast, the smartphone with the PP@G_66_ film showed a uniform temperature distribution on the back without local overheating, with the temperature rising from 23.5 °C to only 32.6 °C, which is 2.8 °C lower than the smartphone without a film and 1.7 °C lower than the one with the commercial PI film (Fig. S17).

To assess the thermal management performance of PP@G_66_ heat sink, we adhered a flexible circuit to it using thermal grease (Fig. 4e), and recorded the temperature changes on the surface of the flexible circuit, as shown in Fig. 4f. The results showed that the PP@G_66_ heat sink exhibited a clearer outline in the thermal infrared images, indicating an ability to dissipate heat faster and more uniformly than the steel heat sink. When switched on, the flexible circuit with PP@G_66_ heat sink shows a much lower surface temperature over time compared with that of the circuits with steel heat sink and without heat sink (Fig. 4g). Once switched off, the cooling behavior of the flexible circuit with PP@G_66_ heat sink was comparable to that of the flexible circuit with steel heat sink (Fig. 4f). Even after 1200 cycles of being switched on and off, the PP@G_66_ nanocomposite heat sink maintained excellent thermal management stability (Fig. 4h).

### Electromagnetic Shielding Performance

**Fig. 5:**
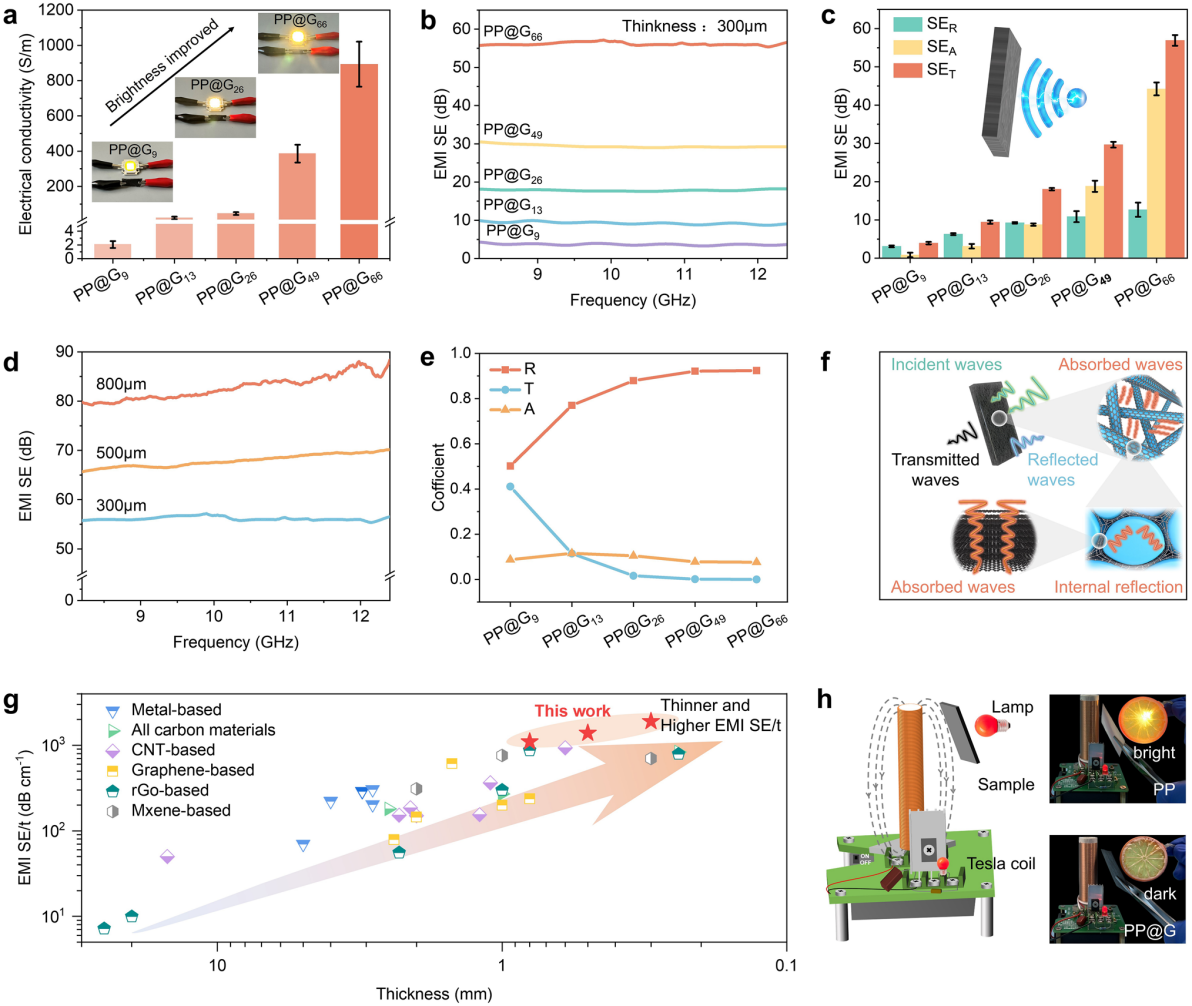
**a** Electrical conductivity of as-prepared PP@G nanocomposites with varying GNP contents; Insets show lighting a LED bulb using as-prepared PP@G nanocomposites with different GNP contents. **b** EMI SE curves and **c** SE_*T*_, SE_*A*_, and SE_*R*_ values of as-prepared PP@G nanocomposites with different GNP contents in X-band. **d** EMI SE curves of as-prepared PP@G_66_ nanocomposites with different thicknesses. **e** Reflectivity (R), absorptivity (A), and transmittance (T) coefficients of as-prepared PP@G nanocomposites with different GNP contents. **f** Schematic illustration of EMI shielding mechanism of as-prepared PP@G nanocomposites. **g** Comparison of EMI SE/t values of as-prepared PP@G nanocomposites with the previously reported materials. **h** Schematic illustration of the shielding effect of as-prepared PP@G nanocomposites and PP film on the electric field generated by the Tesla coil

Generally, a highly continuous electrically conductive network is key to imparting nanocomposites with superior EMI shielding capabilities [41–44]. Therefore, the effect of different GNP contents on the electrical conductivity of PP@G nanocomposites was investigated, as shown in Fig. 5a. When the GNPs content increased from 9 to 66 wt%, the electrical conductivity of the PP@G nanocomposites increased from 2 to 893 S m^−1^. This is because a higher content of GNPs on the PP fiber surface tends to form more continuous and efficient conductive pathways. In the X-band (8.2–12.4 GHz), the EMI shielding value of PP@G nanocomposites (with a thickness of 300 μm) increased to 57 dB as the GNP content increased to 66 wt% (Fig. 5b). When the thickness of the nanocomposite film was 800 μm, the EMI shielding value reached about 88 dB (Fig. 5d), far exceeding the commercial product standard value (30 dB) [45]. Meanwhile, the theoretical EMI SE value (50 dB) of the nanocomposite (Eq. 3) was slightly smaller than the experimental value (57 dB) (Fig. S18), because the presence of unmolten PP fibers increases the nanocomposite's porosity, thereby enhancing electromagnetic wave absorption and the EMI efficiency [46, 47]. Meanwhile, the PP@G nanocomposite exhibits excellent reliability and durability under harsh conditions (Fig. S19). After 100 friction cycle tests, the EMI SE and TC of PP@G remained above 67% and 92%, respectively. Moreover, after being exposed to extreme temperature (−30 and 100 °C) for 24 h, the EMI SE of PP@G increased to 124% and 132%, respectively, while the TC remained at 101% for both temperatures.

The total shielding effectiveness (SE_*T*_), absorption shielding effectiveness (SE_*A*_), and reflection shielding effectiveness (SE_*R*_) were calculated (Eqs. S4-S6) to reveal the high EMI shielding mechanism of PP@G nanocomposites. As shown in Fig. 5c, with increasing GNP contents, both SE_*T*_ and SE_*A*_ of PP@G nanocomposites significantly increased, while SE_*R*_ showed a smaller magnitude of increase compared to SE_*A*_. Meanwhile, the effect of different GNP contents on the reflectivity (R), absorptivity (A), and transmittance (T) of PP@G nanocomposites was calculated (Eqs. S7 and S8), as shown in Fig. 5e. The results show that the R value increases (from 0.5 to 0.9) with increasing GNP content, indicating that reflection plays a dominant role in the EMI shielding [48–51]. As illustrated in Fig. 5f, when electromagnetic waves strike the surface of PP@G nanocomposites, a portion of the waves are directly reflected back into the air due to impedance mismatch between air and GNPs. Subsequently, the remaining electromagnetic waves enter the nanocomposites and interact with the high charge density GNPs, leading to energy dissipation of the electromagnetic waves [52]. Meanwhile, the “core-sheath” structure of PP@G nanocomposites facilitates multiple internal reflections of electromagnetic waves, further dissipating their energy. This synergistic effect of reflection, absorption, and internal re-reflection endows PP@G nanocomposites with high EMI shielding performance.

Figure 5g and Table S6 compare the EMI shielding performance of PP@G nanocomposites with other materials, including Metal-based, All carbon materials, CNT-based, MXene-based, and rGO-based and Graphene-based nanocomposites [53–77]. The results show PP@G nanocomposites exhibit a higher EMI SE/t (the EMI SE value divided by the sample thickness) compared to other reported EMI shielding materials, demonstrating excellent EMI shielding performance.

As shown in Fig. 5h, when the power of a Tesla coil is turned on, a high-frequency electric field is generated. The electromagnetic coupling induces an alternating current in the LED circuit, causing the LED to illuminate. However, when the PP@G nanocomposites (60 mm × 60 mm × 0.8 mm) are inserted between the coil and the LED, their EMI shielding property effectively blocks the electromagnetic field, preventing induced current flow and thus extinguishing the LED, whereas this phenomenon is not observed with PP film. This result highlights the superior EMI shielding capability of PP@G nanocomposites and underscores their potential applications in aerospace, communications, military, and other fields.

Finally, the comprehensive performance of the PP@G was compared with that of previously reported thermal conductive electromagnetic shielding nanocomposites in the literature, as shown in Table S7. The results indicate that the PP@G nanocomposite exhibits significant advantages in several key metrics, including EMI SE/t (1100 dB cm⁻^1^), TC (87 W m⁻^1^ K⁻^1^), cost ($90 ± 5 kg⁻^1^), environmental impact (reduce waste plastic pollution and the generation of micro(nano)plastics) and scalability (170 mm × 170 mm). Thus, it offers strong potential for heat dissipation and EMI shielding applications.

## Conclusions

This work presents an upcycling strategy that transforms waste masks into high-performance TMMs and EMI shielding materials. Because the GNPs were aligned on the PP fiber surface to create highly ordered, continuous, and efficient phonon/electron transport channels, resulting in significantly enhanced specific TCE and reduced R_c_, the prepared PP@G nanocomposite exhibits an outstanding TC of 87 W m⁻^1^ K⁻^1^ and demonstrated excellent heat dissipation capabilities on LED lights and flexible circuits, outperforming commercial PI films and steel heat sinks. Additionally, the PP@G nanocomposite possesses an excellent EMI SE (88 dB) (1100 dB cm^−1^), which can effectively shield against EMI signals. The LCA and TEA results highlight the significant advantages of this upcycling strategy in reducing environmental impacts and economic benefits. This repurposing strategy opens up a new upcycling approach to addressing the plastics waste issue associated with used masks.

## Supplementary Information

Below is the link to the electronic supplementary material.

Supplementary file1 (DOCX 4691 kb)
