# Supplementary material for: Highly Thermal Conductive and Electromagnetic Shielding Polymer Nanocomposites from Waste Masks
Source: Nanomicro Lett. 2025 May 20;17:263. doi: 10.1007/s40820-025-01796-z (PMC12092862; doi:10.1007/s40820-025-01796-z)
Supplement: Supplementary file 1 — Supplementary file1 (DOCX 4691 kb) [file 40820_2025_1796_MOESM1_ESM.docx]

Supporting Information for

**Highly** **Thermal Conductive and Electromagnetic Shielding Polymer Nanocomposites from Waste Masks**

Xilin Zhang^1^, Wenlong Luo^1^, Yanqiu Chen^1^, Qinghua Guo^1^, Jing Luo^2^, Paulomi (Polly) Burey^3^, Yangyang Gao^4^, Yonglai Lu^4^, Qiang Gao^1^*, Jingchao Li^1^*, Jianzhang Li^1*^, and Pingan Song^3^*

^1^ State Key Laboratory of Efficient Production of Forest Resources & MOE Key Laboratory of Wood Material Science and Application, Beijing Forestry University, Beijing 100083, P. R. China

^2^ College of Materials Science and Engineering, Nanjing Forestry University, Longpan Road 159, Xuanwu District, Nanjing 210037, P. R. China

^3^ Centre for Future Materials, School of Agriculture and Environmental Science, University of Southern Queensland Springfield 4300, Australia

^4^ State Key Laboratory of Organic-Inorganic composites, Beijing University of Chemical Technology, Beijing 100029, P. R. China

*Corresponding authors. E-mail: [gaoqiang@bjfu.edu.cn](mailto:gaoqiang@bjfu.edu.cn) (Qiang Gao); [lijingchao@bjfu.edu.cn](mailto:lijingchao@bjfu.edu.cn) (Jingchao Li); [lijzh@bjfu.edu.cn](mailto:lijzh@bjfu.edu.cn) (Jianzhang Li); [pingansong@gmail.com](mailto:pingansong@gmail.com) or [Pingan.song@usq.edu.au](mailto:Pingan.song@usq.edu.au) (Pingan Song)

**S1 Supplemental Experimental Procedures**

**Characterization.** The morphology and hydrophilicity of PP fiber and PP@TA were characterized using a scanning electron microscope equipped with energy-dispersive X-ray spectroscopy (SEM-EDS, Hitachi S4800) and a contact angle goniometer (CAs, Dataphysics OCA20), respectively. The morphology of GNPs was examined using a transmission electron microscope (TEM, FEI Tecnai F20) and an atomic force microscope (AFM, Multimode 8). Raman spectroscopy (Ram, LabRam HR Evolution) was employed to analyze the interactions between PAE and GNPs. Zeta potential measurements of PP fiber, PP@TA, and PAE@GNPs were conducted using a zeta potential analyzer (Malvern Zetasizer Nano ZS90). Functional group changes in PAE, TA, and PAE/TA were investigated using X-ray photoelectron spectroscopy (XPS, Thermo Kalpha). The mass fraction of GNPs in PP@G samples was determined using thermogravimetric analysis (TGA, TA Q50) under a nitrogen atmosphere, with samples heated from 25 to 600 °C at a rate of 10 °C·min⁻¹. The peeling properties, tensile and flexural stress of as-prepared PP@G nanocomposites were measured using an Instron 3366 universal testing machine. The overall thermal conductivities (λ) of as-prepared PP@G nanocomposites were determined using the steady-state heat flow method (ASTM D5470).

The in-plane and out-of-plane thermal conductivities (λ) of as-prepared PP@G nanocomposites were calculated using the following equation:

$\lambda=\alpha\times\rho\times c$ (S1)

where *α*, *ρ*, and *c* represent the thermal diffusivity, density, and specific heat capacity of as-prepared PP@G nanocomposites, respectively. *α* was measured using a flash method thermal conductivity meter (LFA 467 HyperFlash); *ρ* was determined using the water displacement method; and *c* was measured by differential scanning calorimetry (DSC, TA Q2000) using the sapphire method. 3D nano-computed tomography (3D nano-CT) and 2D wide-angle X-ray scattering (2D WAXS) were utilized to analyze the orientation of GNPs in as-prepared PP@G nanocomposites. The specific thermal conductivity enhancements (specific TCE) of as-prepared PP@G nanocomposites were calculated using the following equation:

$Specific TCE=\frac{\lambda-\lambda_{m}}{\lambda_{m}}\times\frac{1}{wt \%}\times100$ (S2)

where *λ* and *λ_m_* are the thermal conductivities of as-prepared PP@G nanocomposites and PP fiber, respectively, and *wt %* is the mass fraction of the thermally conductive filler GNPs in as-prepared PP@G nanocomposites. Thermal infrared images of as-prepared PP@G nanocomposites functioning as cooling substrates and heat sinks were captured using a FLIR E5 camera. The electrical conductivities of as-prepared PP@G nanocomposites were measured using an RTS-8 four-probe tester.

The microwave network vector analyzer (PNA-N5244A) was employed to measure the electromagnetic interference (EMI) shielding effectiveness of as-prepared PP@G nanocomposites in the X-band frequency range (8.2−12.4 GHz) with sample dimensions of 22.9 mm × 10.2 mm.

The theoretical EMI shielding effectiveness of as-prepared PP@G nanocomposites were calculated using Simon's formula:

$EMI SE= 50+10log \left( \sigma/f \right)+1.7t\sqrt{\sigma f}$ (S3)

where *σ* (S/cm) is the electrical conductivities of as-prepared PP@G nanocomposites, *f* (MHz) is the incident microwave frequency, and *t* (cm) is the thickness of as-prepared PP@G nanocomposites. The reflection coefficient S_11_ and transmission coefficient S_21_ were recorded, and the total EMI shielding effectiveness (SE*_T_*), reflection shielding effectiveness (SE*_R_*), absorption shielding effectiveness (SE*_A_*)，and multiple internal reflections shielding effectiveness (SE*_M_*) were calculated using the following equations:

$SE_{T} \left( dB \right) = SE_{R} + SE_{A} + SE_{M}$ (S4) $SE_{R} \left( dB \right)= - 10log \left( 1 - \left| S_{11} \right|^{2} \right)$ (S5)

$SE_{A} (dB) = - 10log [{|S_{21}|}^{2} / (1 - {|S_{11}|}^{2})]$ (S6)

$R= {|S_{11}|}^{2}={|S_{22}|}^{2}$ $T= {|S_{21}|}^{2}={|S_{12}|}^{2}$ (S7)

$R+T+A=1$ (S8)

where SE*_T_*, SE*_R_*, and SE*_A_* represent the total, reflection, and absorption shielding effectiveness, respectively. SE*_M_* represents the multiple internal reflection shielding effectiveness, which can be neglected when SE*_T_* is greater than or equal to 10 dB. R, A, and T are the reflectivity, the absorptivity and the transmittance coefficients, respectively.

**S2 Supplementary Figures and Tables**

**
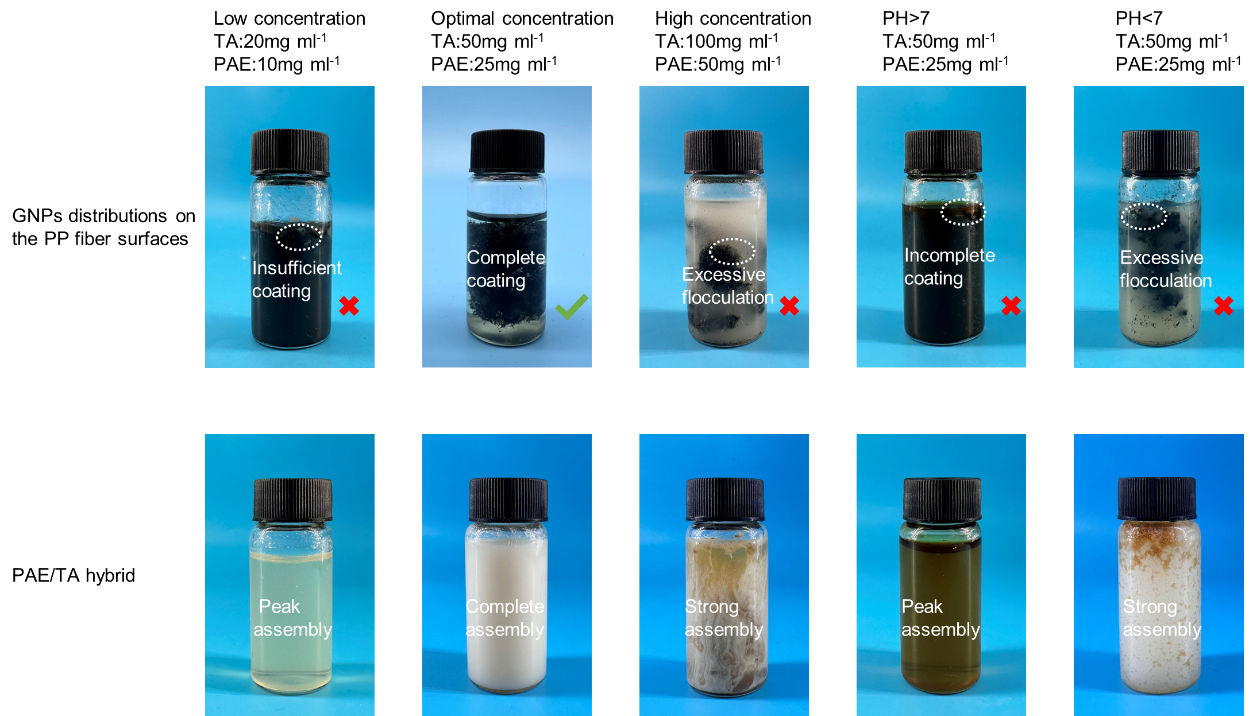
**

**Fig. S1** The distribution of GNPs on the PP fiber surfaces and the state of PAE/TA hybrids under different conditions


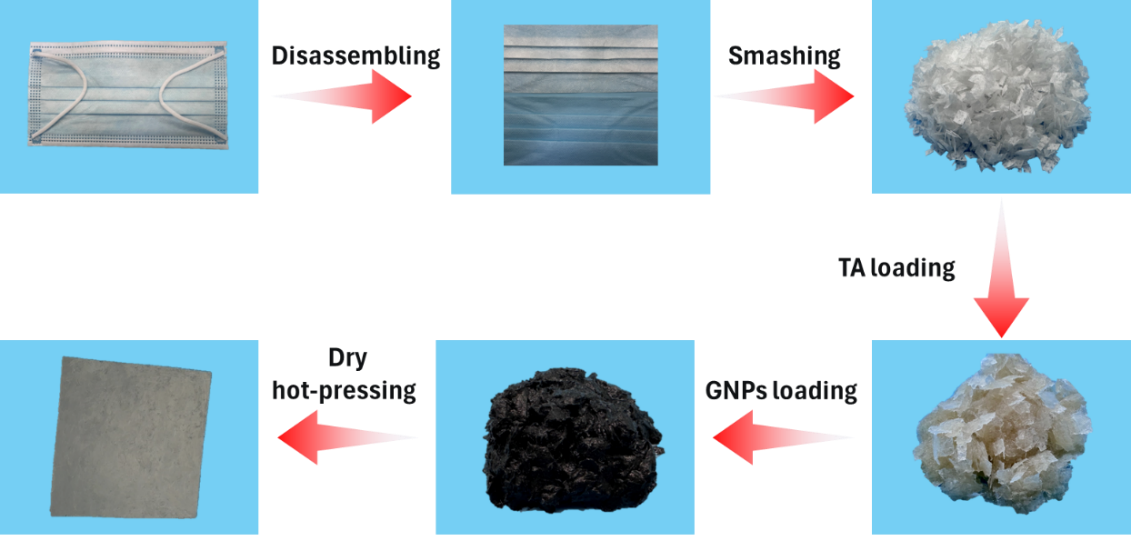


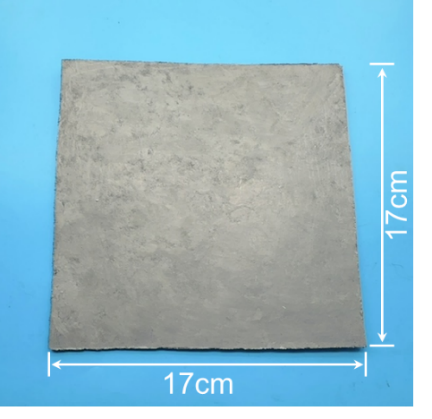


**Fig. S2** The large-scale preparation process and photograph of PP@G nanocomposites (170 mm×170 mm)


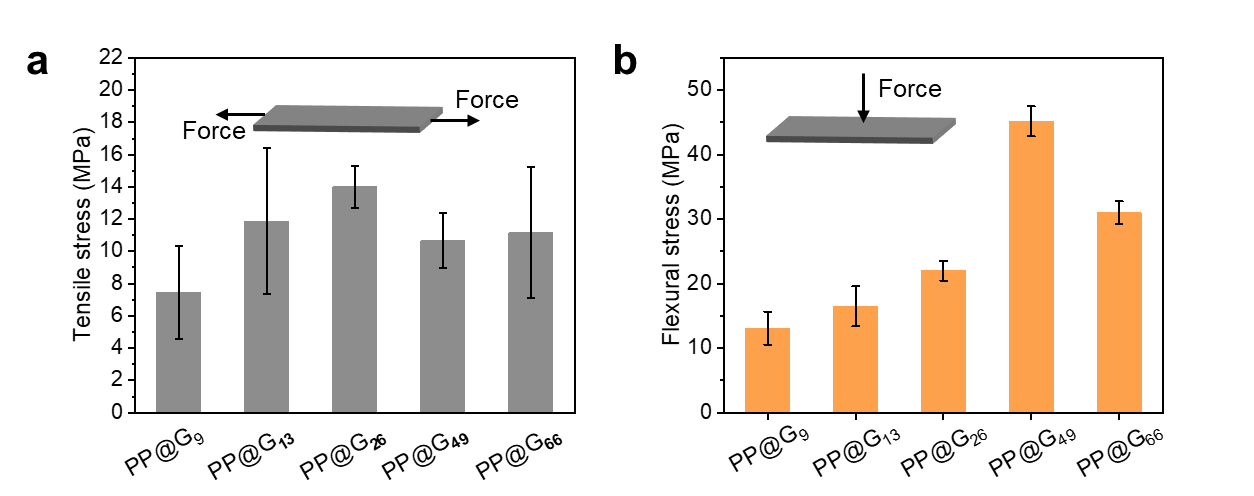


**Fig. S3** **a** Tensile and **b** flexural stress of as-prepared PP@G nanocomposites with different GNP content

**
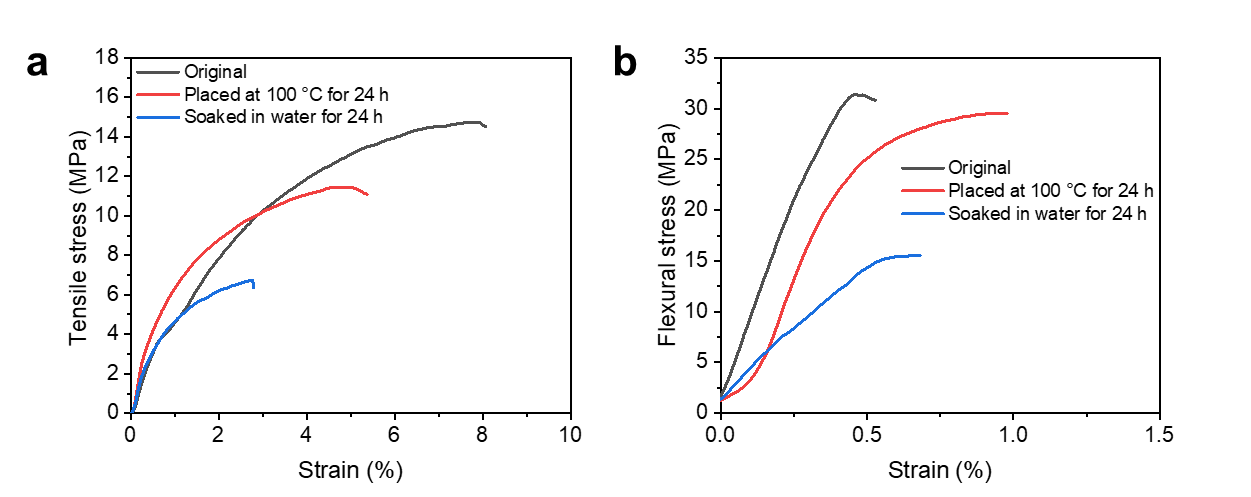
**

**Fig. S4** The variations of **a** the tensile and **b** the flexural stress-strain curves of as-prepared PP@G nanocomposites under extreme conditions

***
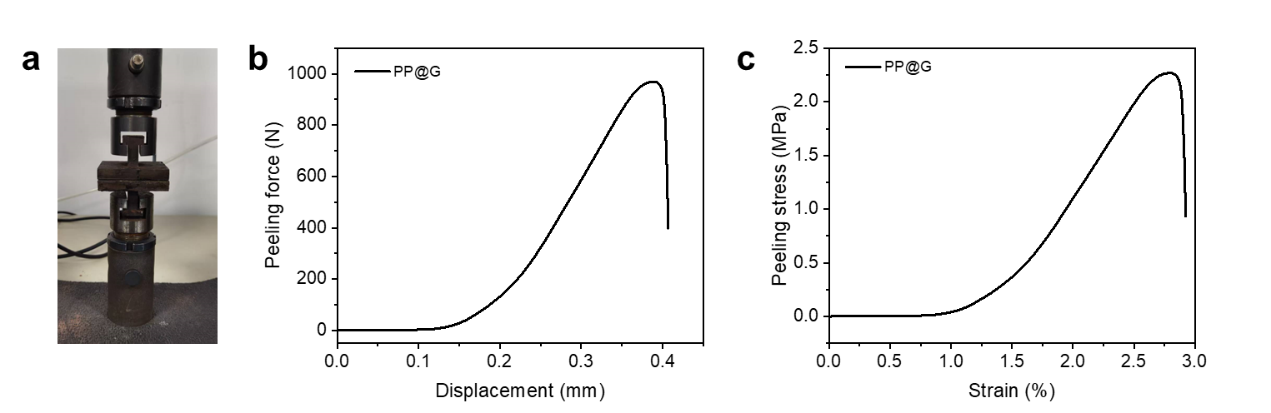
***

**Fig. S5** **a** Photograph of the peeling process of the as-prepared PP@G nanocomposites, along with the corresponding **b** force-displacement and **c** stress-strain curves


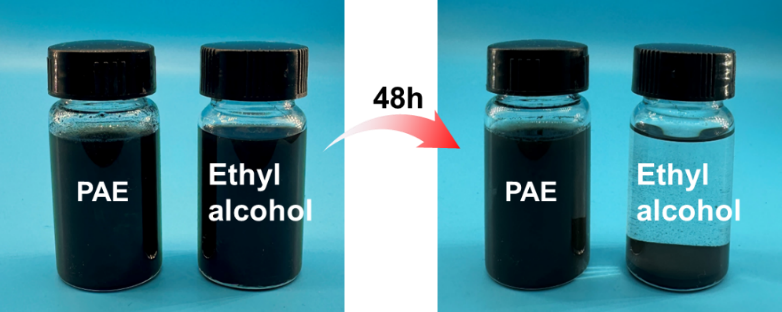


**Fig. S6** Dispersions of GNPs in Ethyl alcohol solution, and aqueous PAE solution before and after being allowed to stand for 48 h


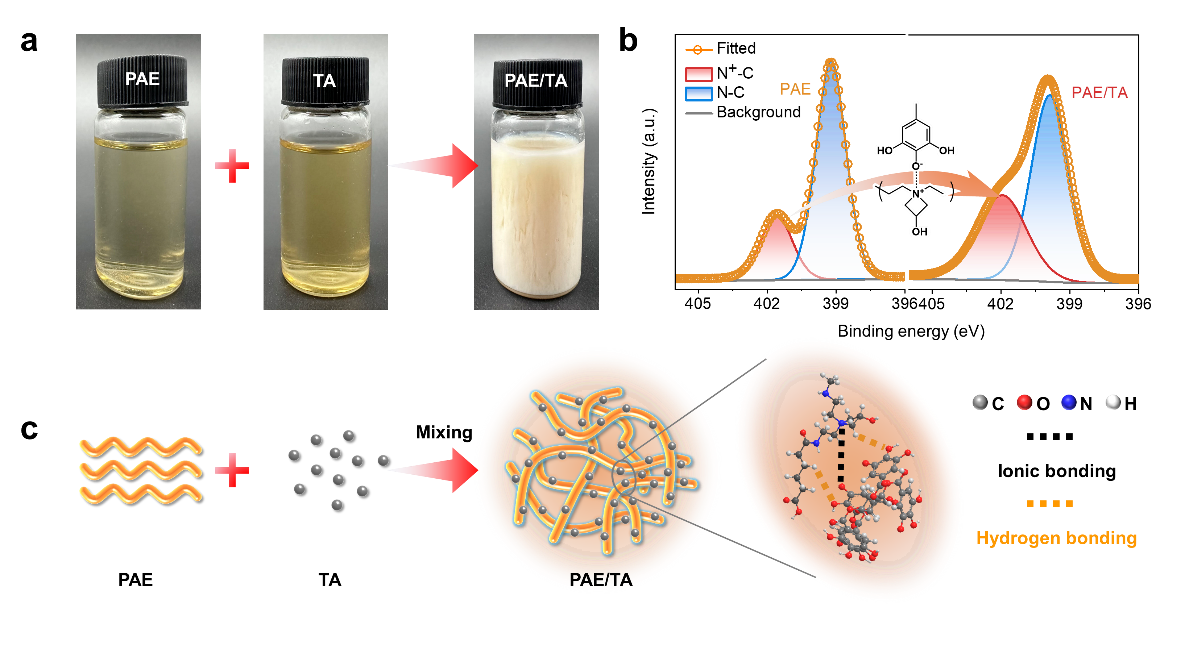


**Fig. S7** Schematic illustration of interactions between PAE and TA


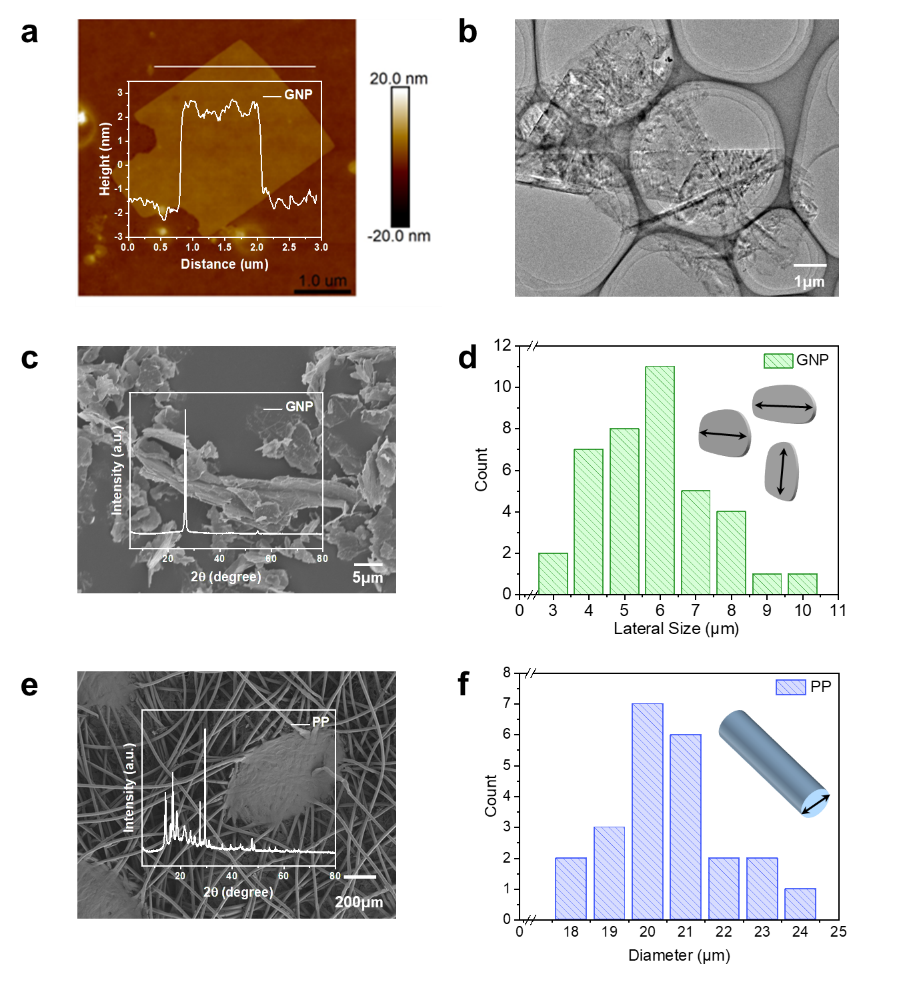


**Fig. S8** **a** AFM image and corresponding thickness measuring curve, **b** typical TEM image, **c** SEM image, XRD, **d** and lateral size distributions of GNPs. **e** SEM image, XRD and **f** Diameter distributions of waste mask PP fibers


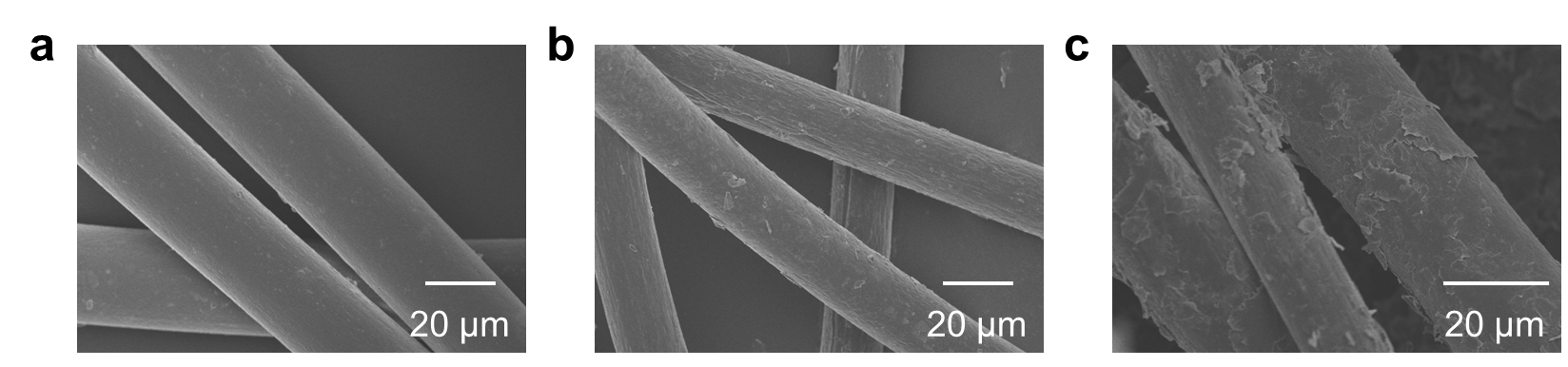


**Fig. S9 a** SEM image of waste mask PP fibers, **b** PP@TA and **c** PP@G


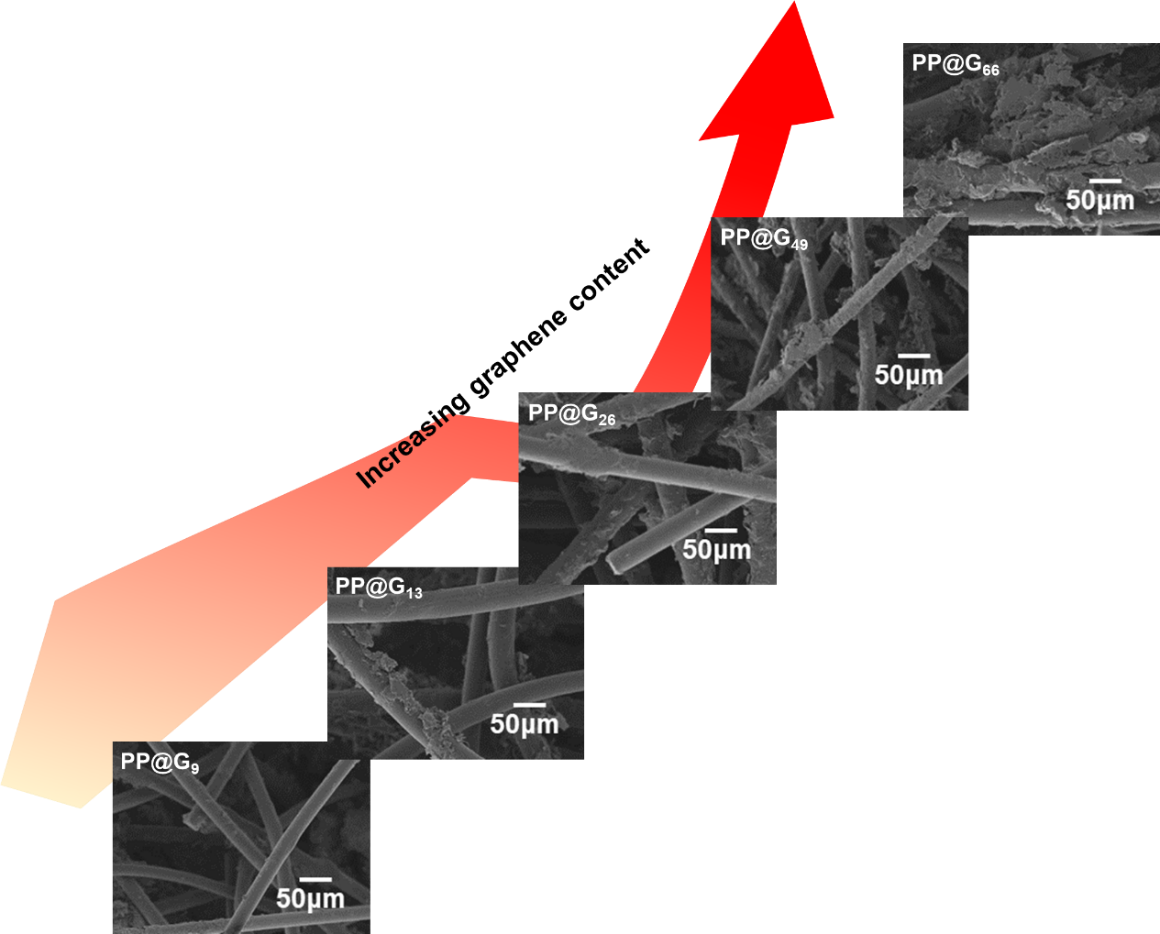


**Fig. S10** SEM images of the PP@GNPs powders with different GNP contents


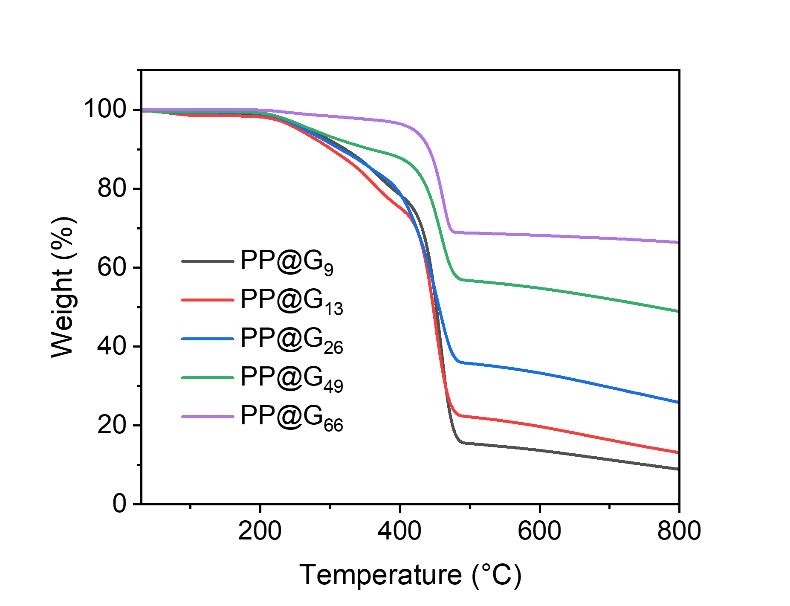


**Fig. S11** TGA curves of as-prepared PP@G nanocomposites with different GNP contents at nitrogen atmosphere

**
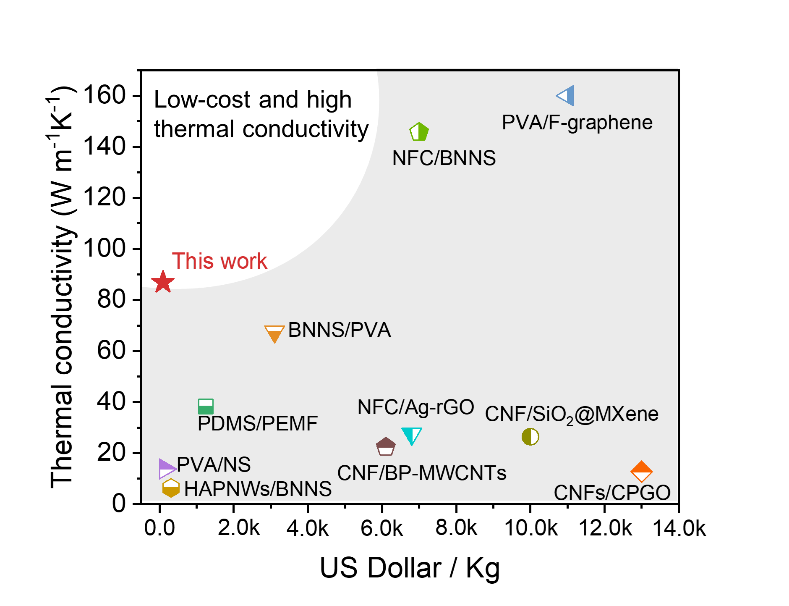
**

**Fig. S12** Comparisons of thermal conductivity and production cost of as-developed PP@G_66_ nanocomposites and previously reported heat-dissipating materials [S1-S10]


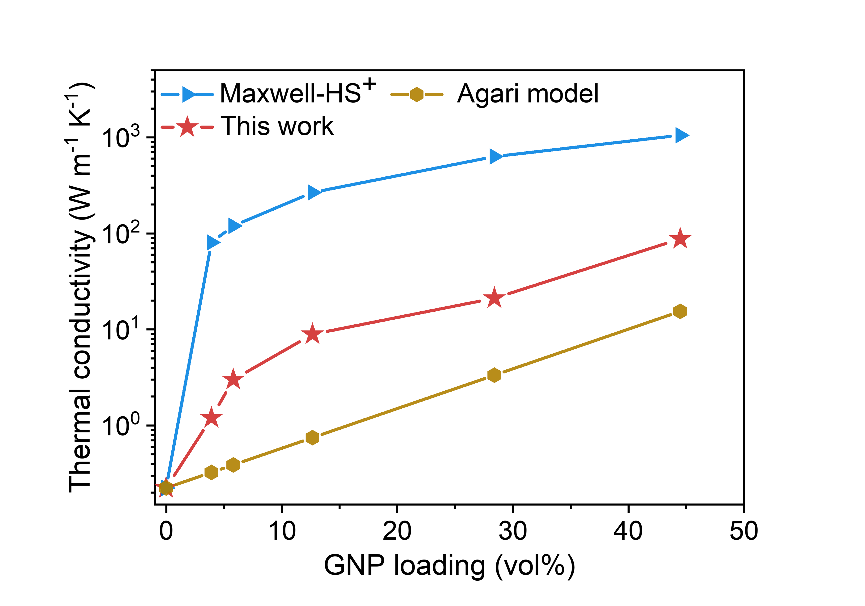


**Fig. S13** Comparison of experimental TC values of as-prepared PP@G nanocomposites and those predicted using Agari and Maxwell-HS^+^ models for different GNP contents


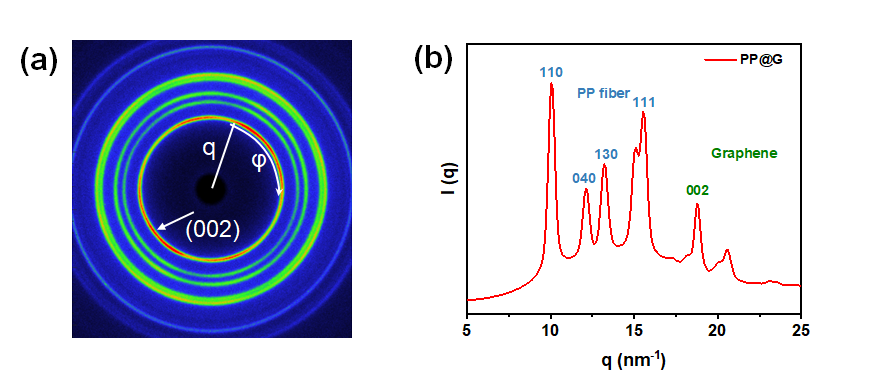


**Fig. S14** 2D WAXS pattern of as-prepared PP@G nanocomposites


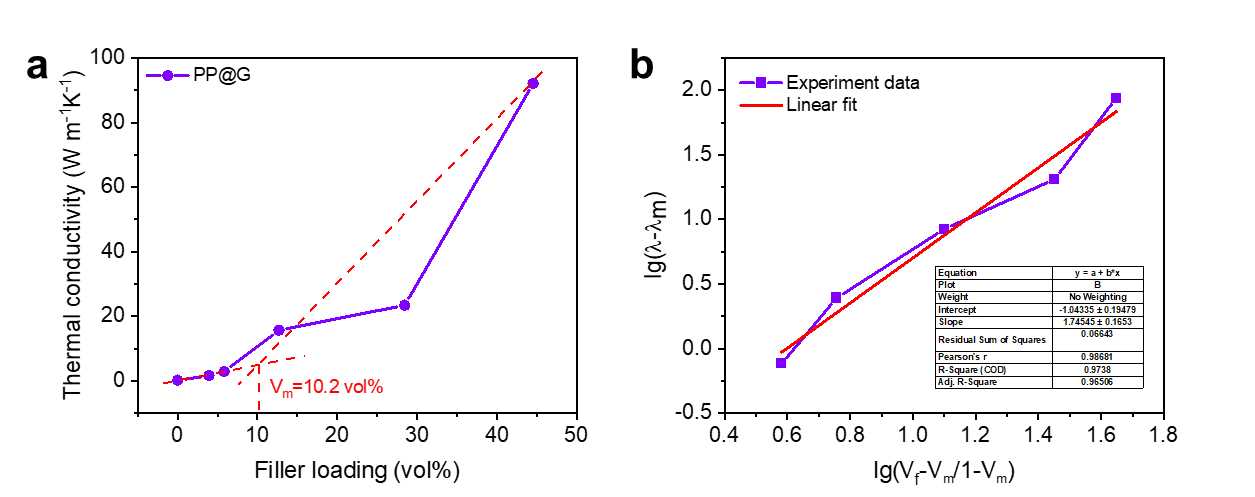


**Fig. S15 a** The tangent process of the experimental data for as-developed PP@G nanocomposites to obtain Vc; and **b** the fitting process by the Foygel model

Here, the Foygel theory was applied to calculate the interfacial thermal resistance at the filler/filler interface, which is expressed as follows：

$\lambda-\lambda_{m}=K_{0}(\frac{V_{f}-V_{m}}{1-V_{m}}$) *^α^*

$$R_{c}=\frac{1}{{K_{0} L (V_{m})}^{\alpha}}$$

In this context, λ and λ*_m_* denote the thermal conductivity of as-developed PP@G_66_ nanocomposites and PP matrix, respectively. K*_0_* refers to a pre-exponential factor ratio that is associated with the contact between GNPs and the topology of the percolation cluster. *α* represents conductivity exponent, while V*_f_* indicates the critical percolation threshold of the fillers. R_c_ is the thermal contact resistance between the GNPs, and *L* signifies the lateral size of the GNPs. For the PP@G composites we developed, fitting the thermal conductivity yielded a calculated R_c_ value of 72,000 K W^-1^ (Fig. S17). Furthermore, the R_c_ values from other previously reported thermal conductive polymer composites with random and 3D networked nanofiller structures were determined using the same methodology.


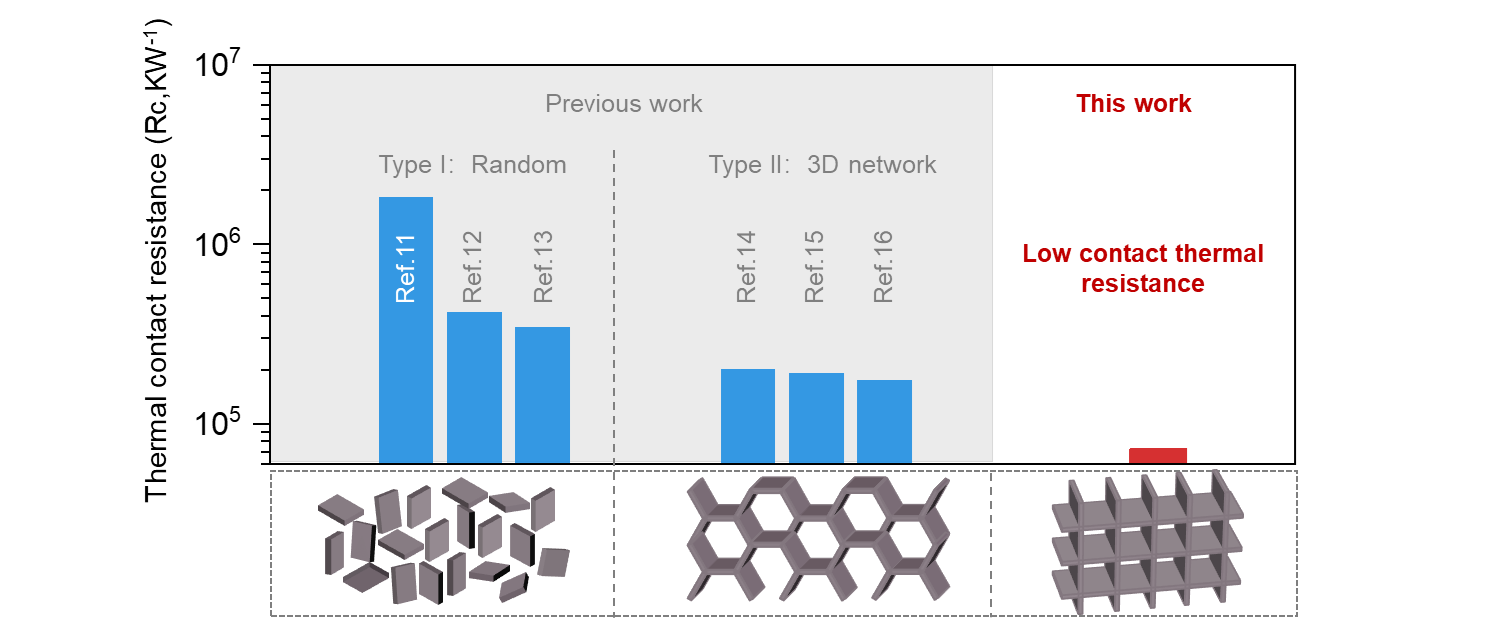


**Fig. S16** Comparison of R_c_ values calculated using the Foygel model in this work with those of previously reported thermal conductive polymer composites with two types of filler distributions [S11-S16]


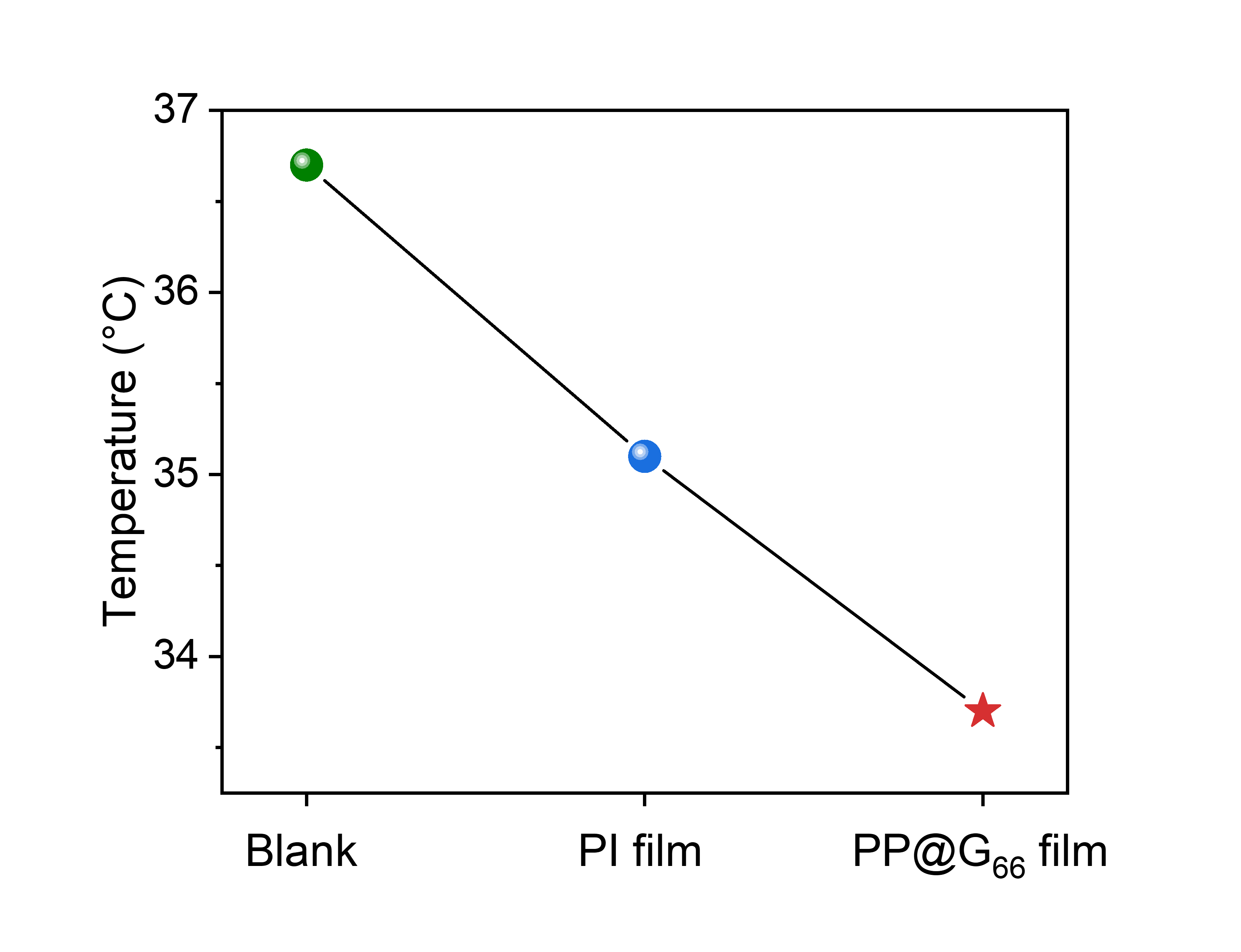


**Fig. S17** The back temperatures of the smartphone using different heat dissipation films after high-load operation for 10 minutes

**Fig. S18** The comparison of experimental and theoretical EMI SE values for as-prepared PP@G_66_ nanocmposites (300 µm thickness)

**
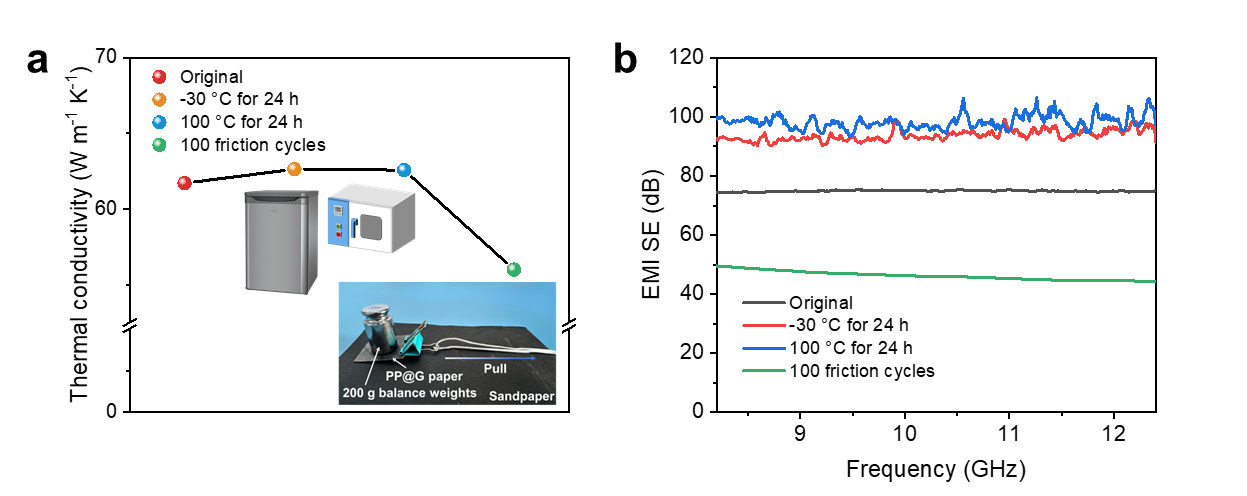
**

**Fig. S19** Changes in **a** TC and **b** EMI SE of the PP@G nanocomposites under different conditions

**Table S1** Environmental impact of using 1 kg of PP for the preparation of PP@G and landfilling

| **Impact category** | **Preparation of PP@G** | **Landfill of PP** | **Reference unit** |
| --- | --- | --- | --- |
| Fossil fuel depletion | -3.47 | 10.80315574 | MJ surplus |
| Global warming | -0.351 | 2.527030755 | kg CO_2_ eq |
| Carcinogenics | 3.64E-09 | 8.84082E-08 | CTUh |
| Acidification | 0.000375 | 0.00884379 | kg SO_2_ eq |
| Ecotoxicity | 1.02 | 7.813904035 | CTUe |
| Smog | 0.0149 | 0.113794478 | kg O_3_ eq |
| Eutrophication | 0.000804 | 0.003387112 | kg N eq |
| Ozone depletion | 1.61E-08 | 6.57458E-08 | kg CFC-11 eq |
| Non carcinogenics | 7.17E-08 | 1.69754E-07 | CTUh |
| Respiratory effects | 0.000504 | 0.00082594 | kg PM2.5 eq |

**Table S2** Comparisons of thermal conductivity and fabrication cost of PP@G_66_ nanocomposites and previously reported heat-dissipating materials

|  | **Fillers** | **Matrix types** | **Contents (wt.%)** | **Thermal conductivity**  **(****W m^-1^ K^-1^)** | **Fabrication cost**  **(Dollar/kg)** |
| --- | --- | --- | --- | --- | --- |
| **Previously**  **reported** | F-graphene | PVA | 93 | 61.3 | 11000 ± 550 |
|  | BNNS | NFC | 50 | 145.7 | 7000 ± 350 |
|  | BNNS | PVA | 83 | 67.6 | 3100 ± 155 |
|  | BNNS | HAPNWs | 30 | 6.4 | 310 ± 15.5 |
|  | BP-MWCNTs | CNF | 20 | 22.38 | 6100 ± 305 |
|  | PEMF | PDMS | 55.08 | 38.27 | 1250 ± 62.5 |
|  | NS | PVA | 10 | 13.8 | 150 ± 7.5 |
|  | Ag-rGO | NFC | 9.6 | 27.55 | 6800 ± 340 |
|  | SiO_2_@MXene | CNF | 97.5 | 26.4 | 10000 ± 500 |
|  | CPGO | CNFs | 70 | 12.75 | 13000 ± 650 |
|  | GNPs | PVA | 60.01 | 82.4 | 258 ± 12.9 |
| **This work** | GNP | PP | 66 | 87 | 90±5 |

**Table S3** Comparison of TC for PP@G nanocomposites using the steady-state heat flow method (ASTM D5470) and the laser flash method (out-of-plane)

| **GNP content (wt%)** | **Thermal conductivity** **(W m^-1^ K^-1^)** | |
| --- | --- | --- |
|  | The steady-state  heat flow method | the laser flash method  (out-of-plane) |
| 9 | 0.3 | 0.3 |
| 13 | 0.6 | 0.4 |
| 26 | 1.6 | 0.5 |
| 49 | 3.1 | 1.8 |
| 66 | 3.1 | 3.4 |

**Table S4** Summary of thermal conductivity models of composites

| **Models** | **Effective TC equation** |
| --- | --- |
| Agari | $\log\lambda=Vk_{2}\log\lambda_{f}+\left( 1-V \right)\log(k_{1}\lambda_{m})$ |
| Maxwell-HS^+^ | $\lambda=\lambda_{f}\frac{2\lambda_{f}+\lambda_{m}-2\left( 1-V \right)(\lambda_{f}-\lambda_{m})}{2\lambda_{f}+\lambda_{m}+\left( 1-V \right)(\lambda_{f}-\lambda_{m})}$ |

* In the above model, λ represents the effective thermal conductivity of composites, λ_m_ represents the thermal conductivity of matrix in the composite, λ_f_ represents the thermal conductivity of filler in the composite, and V represents the volume fraction of the filler. In the Agari model, K_1_ is an indicator that measures the influence of filler on the secondary structure of the polymer matrix, such as crystallinity, crystal size, etc., and it is related to the changes in the thermal conductivity of the polymer matrix. K_2_ measures the ease with which filler form conducting chains.

**Table S5** Comparisons of in-plane specific TCE along the preferred direction of heat transfer between as-prepared PP@G_66_ nanocomposites and other polyolefin composites filled with two types of nanofiller structure. λ_m_: Thermal conductivity of matrix; λ: Thermal conductivity of composites; CNTs: Carbon nanotubes

| **Fillers** | | **Matrix** | **λ_m_** | **λ** | **Content (wt%)** | **Specific TCE** | **Refs.** |
| --- | --- | --- | --- | --- | --- | --- | --- |
|  |  |  | **(W m^-1^ K^-1^)** | |  |  |  |
| **Type I：**  **Random）** | Boron nitride + CNTs | PE | 0.38 | 1.79 | 50 | 7 | [36] |
|  | Boron nitride | PP | 0.18 | 0.72 | 29 | 10 | [37] |
|  | CNTs | PP | 0.2 | 0.55 | 16 | 11 | [38] |
|  | Graphene | PP | 0.2 | 0.67 | 15 | 16 | [39] |
|  | Graphene | PE | 0.3 | 2.91 | 40 | 22 | [40] |
| **Type II：**  **3D network** | Graphene | PP | 0.2 | 0.86 | 16.7 | 20 | [41] |
|  | Graphene | PE | 0.4 | 4.13 | 33 | 28 | [42] |
|  | Graphene | PE | 0.33 | 1.84 | 10 | 46 | [43] |
|  | Graphene | PP | 0.22 | 1.53 | 10 | 60 | [43] |
|  | Graphene | PP | 0.74 | 1.58 | 1.83 | 63 | [44] |
| **This work** | Graphene | PP | 0.2 | 87 | 66 | 660 | **/** |

**Table S6** Comparisons of the EMI shielding performance of PP@G nanocomposites with other EMI composite materials

| **Type** | **Materials** | **Thickness**  **(mm)** | **EMI SE**  **(dB)** | **SMI SE/t**  **(dB cm^-1^)** | **Refs.** |
| --- | --- | --- | --- | --- | --- |
| Metal-based | Copper | 3.1 | 90 | 290.3 | [52] |
|  | Stainless steel | 4 | 89 | 222.5 | / |
|  | Ni fiber/PES | 2.85 | 58 | 203.5 | [53] |
|  | Ni filaments | 2.85 | 87 | 305.3 |  |
|  | AgNW/PI | 5 | 35 | 70 | [54] |
| All carbon  materials | CNT sponge | 0.24 | 20 | 833.3 | [55] |
|  | CNT/Graphene | 1 | 27 | 270 | [56] |
|  | rGO | 2.5 | 45.1 | 180.4 | [67] |
| CNT based | CNTs/PC | 2.1 | 39 | 185.7 | [57] |
|  | MWCNTs/PS | 2 | 30 | 150 | [58] |
|  | SWCNTs/PS | 1.2 | 18.5 | 154.2 | [59] |
|  | MWCNTs/WPU | 2.3 | 35 | 152.17 | [60] |
|  | MWCNTs/ABS | 1.1 | 40 | 363.6 | [61] |
|  | MWCNT/GF/PDMS | 15 | 75 | 50 | [62] |
|  | MWCNT-based composite paper | 0.6 | 56 | 933.3 | [63] |
| MXene based | MXene/PVA | 0.3 | 21 | 700 | [64] |
|  | MXene/PS | 2 | 62 | 310 | [65] |
|  | MXene/wax | 1 | 76.1 | 761 | [66] |
| rGO based | rGO/PS | 2.5 | 45.1 | 180.4 | [67] |
|  | rGO/PU | 20 | 19.9 | 9.95 | [68] |
|  | rGO/PDMS | 1 | 30 | 300 | [73] |
|  | rGO/PMMA | 0.24 | 19 | 791.7 | [75] |
|  | rGO/PEI | 2.3 | 12.8 | 55.7 | [69] |
|  | rGO/Fe_3_O_4_/PEI | 25 | 18 | 7.2 | [70] |
|  | rGO/PEDOT | 0.8 | 70 | 875 | [71] |
| Graphene-  based | Graphene/PEDOT:PS S | 1.5 | 91.9 | 612.7 | [72 |
|  | Graphene/PDMS | 1 | 20 | 200 | [73] |
|  | Graphene/PS | 2 | 29 | 145 | [74] |
|  | Graphene/PMMA | 2.4 | 19 | 79.2 | [75] |
|  | Graphene/PI | 0.8 | 19 | 237.5 | [76] |
| This work | GNP/PP | 0.8 | 88 | 1100 | / |
|  | GNP/PP | 0.5 | 69 | 1380 | / |
|  | GNP/PP | 0.3 | 57 | 1900 | / |

**Table S7** Comparisons of comprehensive performance of our strategy and previously reported thermal conductive electromagnetic shielding composites

| **EMI SE** | **EMI SE/t** | **Thermal Conductivity**  **(W m^-1^ K^-1^)** | **Cost ($/kg)** | **Scalability (mm)** | **Environmental**  **benefits** | **Refs.** |
| --- | --- | --- | --- | --- | --- | --- |
| **(dB cm^-1^)** | |  |  |  |  |  |
| 67.86 | 339.3 | / | / | 17.5×17.5 | / | [S17] |
| 61.4 | 245.6 | 2.187 | / | 21×21 | / | [S18] |
| / | / | 69.74 | / | 10×10 | / | [S19] |
| / | / | 67.6 | 310 ± 155 | 44×44 | / | [S20] |
| 88 | 1100 | 87 | 90 ± 5 | 170×170  (>150) | 1.Reduce waste plastic pollution;  2. Reduce the generation of micro(nano)plastics | This  work |

**Supplementary References**

1. L. Cheng, J. Feng, Flexible and fire-resistant all-inorganic composite film with high in-plane thermal conductivity. Chem. Eng. J. **398**, 125633 (2020). <https://doi.org/10.1016/j.cej.2020.125633>
2. Y. Gao, F. Müller-Plathe, Increasing the thermal conductivity of graphene-polyamide-6, 6 nanocomposites by surface-grafted polymer chains: calculation with molecular dynamics and effective-medium approximation. J. Phys. Chem. B **120**(7), 1336–1346 (2016). <https://doi.org/10.1021/acs.jpcb.5b08398>
3. Z. Qu, K. Wang, C.-A. Xu, Y. Li, E. Jiao et al., Simultaneous enhancement in thermal conductivity and flame retardancy of flexible film by introducing covalent bond connection. Chem. Eng. J. **421**, 129729 (2021). <https://doi.org/10.1016/j.cej.2021.129729>
4. D. Suh, C.M. Moon, D. Kim, S. Baik, Ultrahigh thermal conductivity of interface materials by silver-functionalized carbon nanotube phonon conduits. Adv. Mater. **28**(33), 7220–7227 (2016). <https://doi.org/10.1002/adma.201600642>
5. Q. Yan, W. Dai, J. Gao, X. Tan, L. Lv et al., Ultrahigh-aspect-ratio boron nitride nanosheets leading to superhigh in-plane thermal conductivity of foldable heat spreader. ACS Nano **15**(4), 6489–6498 (2021). <https://doi.org/10.1021/acsnano.0c09229>
6. S. Yang, B. Xue, Y. Li, X. Li, L. Xie et al., Controllable Ag-rGO heterostructure for highly thermal conductivity in layer-by-layer nanocellulose hybrid films. Chem. Eng. J. **383**, 123072 (2020). <https://doi.org/10.1016/j.cej.2019.123072>
7. Y. Zhan, B. Nan, Y. Liu, E. Jiao, J. Shi et al., Multifunctional cellulose-based fireproof thermal conductive nanocomposite films assembled by *in situ* grown SiO_2_ nanoparticle onto MXene. Chem. Eng. J. **421**, 129733 (2021). <https://doi.org/10.1016/j.cej.2021.129733>
8. Y. Zhang, C. Lei, K. Wu, Q. Fu, Fully organic bulk polymer with metallic thermal conductivity and tunable thermal pathways. Adv. Sci. **8**(14), 2004821 (2021). <https://doi.org/10.1002/advs.202004821>
9. Y. Zhuang, K. Zheng, X. Cao, Q. Fan, G. Ye et al., Flexible graphene nanocomposites with simultaneous highly anisotropic thermal and electrical conductivities prepared by engineered graphene with flat morphology. ACS Nano **14**(9), 11733–11742 (2020). <https://doi.org/10.1021/acsnano.0c04456>
10. H. Zhu, Y. Li, Z. Fang, J. Xu, F. Cao et al., Highly thermally conductive papers with percolative layered boron nitride nanosheets. ACS Nano **8**(4), 3606–3613 (2014). <https://doi.org/10.1021/nn500134m>
11. Y. Seki, B. Avci, S. Uzun, N. Kaya, M. Atagur et al., The using of graphene nano-platelets for a better through-plane thermal conductivity for polypropylene. Polym. Compos. **40**(S2), E1320–E1328 (2019). <https://doi.org/10.1002/pc.24979>
12. M. Saeidijavash, J. Garg, B. Grady, B. Smith, Z. Li et al., High thermal conductivity through simultaneously aligned polyethylene lamellae and graphene nanoplatelets. Nanoscale **9**(35), 12867–12873 (2017). <https://doi.org/10.1039/C7NR04686C>
13. F. Tarannum, R. Muthaiah, R.S. Annam, T. Gu, J. Garg, Effect of alignment on enhancement of thermal conductivity of polyethylene-graphene nanocomposites and comparison with effective medium theory. Nanomaterials **10**(7), 1291 (2020). <https://doi.org/10.3390/nano10071291>
14. F. Xiao, F. Weng, D. Guo, E. Koranteng, H. Zhou et al., Preparation and characterization of thermosetting wood flour composite material with high biomass content. Polym. Compos. **42**(9), 4822–4830 (2021). <https://doi.org/10.1002/pc.26192>
15. W. Guo, G. Chen, Fabrication of graphene/epoxy resin composites with much enhanced thermal conductivity *via* ball milling technique. J. Appl. Polym. Sci. **131**(15), 40565 (2014). <https://doi.org/10.1002/app.40565>
16. C.-C. Teng, C.M. Ma, C.-H. Lu, S.-Y. Yang, S.-H. Lee et al., Thermal conductivity and structure of non-covalent functionalized graphene/epoxy composites. Carbon **49**(15), 5107–5116 (2011). <https://doi.org/10.1016/j.carbon.2011.06.095>
17. Q. Guo, H. Tian, Y. Cheng, S. Wang, Z. Li et al., Structural-functional integrated graphene-skinned aramid fibers for electromagnetic interference shielding. ACS Nano **18**(49), 33566–33575 (2024). <https://doi.org/10.1021/acsnano.4c11782>
18. S. Shi, Y. Jiang, H. Ren, S. Deng, J. Sun et al., 3D-printed carbon-based conformal electromagnetic interference shielding module for integrated electronics. Nano-Micro Lett. **16**(1), 85 (2024). <https://doi.org/10.1007/s40820-023-01317-w>
19. Z. Ma, J. Wang, Z. Hao, J. Dai, X. Zhu et al., Novel graphene-epoxy composite with aligned architecture and ultrahigh thermal conductivity. Adv. Funct. Mater. **35**(2), 2412534 (2025). <https://doi.org/10.1002/adfm.202412534>
20. Q. Yan, W. Dai, J. Gao, X. Tan, L. Lv et al., Ultrahigh-aspect-ratio boron nitride nanosheets leading to superhigh in-plane thermal conductivity of foldable heat spreader. ACS Nano **15**(4), 6489–6498 (2021). <https://doi.org/10.1021/acsnano.0c09229>
